# Phytochemistry and Wound-Healing, Enzyme-Inhibitory, and Antifungal Activities of the Wild Forage Legume *Lotus rectus* L.

**DOI:** 10.3390/plants15091367

**Published:** 2026-04-29

**Authors:** Manuel González-Vázquez, Ana Quílez Guerrero, Mónica Zuzarte, Lígia Salgueiro, Jorge Alves-Silva, Rocío De la Puerta

**Affiliations:** 1 Department of Pharmacology, Faculty of Pharmacy, University of Seville, 41012 Seville, Spain; mgonzalez15@us.es (M.G.-V.); quilez@us.es (A.Q.G.); 2 Faculty of Pharmacy, University of Coimbra, Azinhaga de S. Comba, 3000-548 Coimbra, Portugal; mzuzarte@uc.pt (M.Z.); ligia@ff.uc.pt (L.S.); jmasilva@ff.uc.pt (J.A.-S.); 3 Faculty of Medicine, Coimbra Institute for Clinical and Biomedical Research, Centre for Innovative Biomedicine and Biotechnology (iCBR-CIBB), University of Coimbra, Azinhaga de S. Comba, 3000-548 Coimbra, Portugal; 4 Department of Chemical Engineering, Chemical Engineering and Renewable Resources for Sustainability (CERES), University of Coimbra, 3030-790 Coimbra, Portugal

**Keywords:** collagenase, dermatophytes, flavonols, *Lotus rectus*, proanthocyanidins, reactive oxygen species, tyrosinase, wound-healing, xanthine oxidase

## Abstract

*Lotus rectus* L. is an underexplored forage legume with reported traditional uses in skin-related conditions. This study aimed to characterize the phytochemical profile of its aqueous leaf extract (LRAE) and to explore its bioactivity in vitro. Phytochemical characterization was carried out using spectrophotometric assays and UHPLC-HRMS/MS. Cytocompatibility was assessed by the resazurin assay in HaCaT keratinocytes and NIH/3T3 fibroblasts, while wound-healing potential was evaluated using a scratch assay. Enzyme inhibitory activities (xanthine oxidase, collagenase, hyaluronidase, and tyrosinase) were determined spectrophotometrically. Antioxidant capacity was assessed using chemical assays (DPPH and ABTS), biologically relevant reactive oxygen species, and metal chelation assays. Antifungal activity was evaluated against clinically relevant yeasts and dermatophytes using standardized macrodilution methods. LRAE showed a relatively high content of flavonoids and proanthocyanidins, particularly flavonol glycosides. The extract was cytocompatible at all tested concentrations and showed an increased closure of the scratched area in vitro. It exhibited antioxidant activity and inhibited xanthine oxidase, while more moderate effects were observed for collagenase and tyrosinase, and minimal activity was detected against hyaluronidase. Antifungal activity was limited, with modest effects observed only against selected dermatophytes at high concentrations. Overall, these findings provide preliminary in vitro evidence of bioactivity associated with the traditional use of this species, supporting further investigation to better characterize the biological relevance of this understudied species.

## 1. Introduction

The skin is the largest organ of the human body and serves as a versatile barrier against pathogens, injury, and ultraviolet radiation. Consequently, wound healing is a complex biological process, involving coordinated mechanisms that ensure tissue repair and closure [1]. This process comprises sequential and overlapping phases: immediate haemostasis, followed by inflammation, proliferation and remodeling. In healthy individuals, the entire process typically lasts 4–6 weeks until complete recovery of the affected area [2]. However, the presence of certain comorbidities such as immune cells, hypoxia, and persistent infections can promote a pro-inflammatory microenvironment. This prevents progression to the proliferative phase and may lead to chronic wounds that remain unhealed for extended periods [3,4]. This environment is characterized by the persistence of neutrophils and inflammatory mediators, along with elevated oxidative stress associated with reactive oxygen and nitrogen species, partly driven by xanthine oxidase, a molybdoflavin enzyme involved in purine metabolism. Moreover, it is further marked by excessive extracellular matrix (ECM) degradation mediated by overexpressed matrix metalloproteinases (e.g., collagenase) and other ECM-degrading enzymes (e.g., hyaluronidase) [3,5,6]. Chronic wounds are estimated to affect 1–2% of the global population during their lifetime and currently impose a socioeconomic burden comparable to that of other major chronic diseases [3,7]. In this context, chronic wounds are frequently associated with microbial colonization, including dermatophytes and *Candida* species, which can further impair the healing process [8].

In this context, the investigation of medicinal plants traditionally used to treat wounds, ulcers, or burns may represent an effective strategy for advancing the treatment of this and other skin disorders [9]. Moreover, the study of such plants can serve as a proxy for bioactivity screening, often leading to the identification of extracts with antimicrobial, antioxidant, and enzyme-inhibitory activities relevant to wound healing and other skin disorders [10].

*Lotus rectus* L. (syn. *Dorycnium rectum* (L.) Ser.) is a perennial leguminous herb also known as greater badassi or erect canary clover, native to the Mediterranean region and recently introduced to southern Australia. In the Iberian Peninsula it is commonly known as hierba junciana (Spain) or erva-salsicheira (Portugal), where its leaf decoction has traditionally been used topically and orally as a treatment for skin infections, eczema, wounds or stomach ulcers. It has also been used in ethnoveterinary for burns, wounds and external parasitic infections [11,12,13]. In addition, the species has traditionally been used as a wild forage legume for livestock in Spain and Italy, while in Australia and New Zealand it has been introduced and extensively investigated for its potential as a forage crop [13,14,15]. Indeed, its use as a forage legume has attracted considerable interest due to its gastrointestinal anthelmintic activity in livestock, mainly attributed to the presence of condensed tannins in the aerial parts of the plant [16]. Interestingly, the wound-healing properties of condensed tannins are well recognized, in line with the traditional medicinal uses of this species [17,18]. However, beyond its investigation as a forage legume, the bioactive potential of this species in relation to its traditional uses for skin conditions remains largely unexplored. In contrast, apart from its well-established relevance in plant biology, other *Lotus* species have been studied from a phytochemical and pharmacological perspective in relation to their anti-inflammatory, antimicrobial, and antioxidant activities, leading to promising results in these areas [19,20,21]. Indeed, the genus *Lotus* is currently attracting increasing interest as a source of innovation, with examples such as the leaf extract of *L. maritimus*, which has been incorporated as an active ingredient in cosmetic products, and biotechnological approaches based on *L. japonicus* cell cultures, which have demonstrated the ability to stimulate extracellular matrix production in human dermal fibroblasts [22,23]. In this context, considering its traditional uses, the presence of condensed tannins in the aerial parts of the plant, and the bioactive potential reported in other *Lotus* species, the present study was designed to explore this ethnopharmacological background. Specifically, we aimed to characterize the phenolic composition of LRAE and to evaluate its in vitro effects on processes related to skin function, including scratch closure, enzyme inhibition, antioxidant activity, and antifungal effects.

## 2. Results

### 2.1. Phenolic Composition of the Aqueous Leaf Extract of L. rectus

#### 2.1.1. Extraction Yield and Determination of Major Phenolic Groups

The extraction yield of LRAE was 25.90 ± 1.97% (*w*/*w*), expressed as mean ± SD (*n* = 3 extractions). The extract showed a moderate phenolic content, corresponding to approximately 8% (*w*/*w*) of dry extract (Table 1). Its phytochemical profile was characterized by substantial levels of flavonoids and proanthocyanidins. Consistent with the high proanthocyanidin content, the tannin assay also indicated a considerable proportion of condensed tannins in the extract, while hydroxycinnamic acids were not detected.

#### 2.1.2. Identification of Phenolic Compounds by Ultra-High-Pressure Liquid Chromatography Coupled with High-Resolution Tandem Mass Spectrometry (UHPLC-HRMS/MS)

Phenolic profiling of LRAE by UHPLC-HRMS/MS identified various phenolic compounds represented in Table 2, including glycosylated flavonols (compounds **1**, **2**, **6** and **8**), flavanones (compounds **9** and **10**), flavones (compound **11**), flavan-3-ols (compound **4**), hydroxybenzoic acids (compounds **3** and **7**) and hydroxycinnamic acids (compound **5**). All compounds were identified using an internal database of phenolic compounds provided by the spectrometry service of the Centro de Investigación y Transferencia of the University of Seville (CITIUS), as detailed in Section 4.4 of this article. Additional information on the identified compounds is provided in the Supplementary Materials, including an expanded version of Table 2, with retention time deviations and MS/MS fragment mass errors (Supplementary Table S1), as well as representative chromatographic and spectral data compared with reference standards (Supplementary Document S1).

Regarding quantitative analysis, a significant amount of kaempferol-3,7-O-α-di-rhamnopyranoside (5.60 ± 0.006 mg/g dry extract) was detected, whereas quercetin-3-O-galactoside (hyperoside) was present in a comparatively lower concentration (Table 2). Additional compounds evaluated for quantification included the flavanone naringenin and the hydroxycinnamic acid caffeic acid. However, although both compounds were detected above the limit of detection (LOD), their concentrations did not exceed the limit of quantification (LOQ) . Therefore, the content of both compounds is reported in Table 2 as <LOQ. The quantitative results obtained for caffeic acid, together with the lack of detection of other hydroxycinnamic acids by UHPLC-HRMS/MS, are consistent with the results obtained in the total hydroxycinnamic acids assay (Table 1), suggesting that LRAE does not contain significant amounts of this class of compounds.

### 2.2. Effect of the Aqueous Leaf Extract of L. rectus on Cell Viability of HaCaT Keratinocytes and NIH/3T3 Fibroblasts

**Figure 1:**
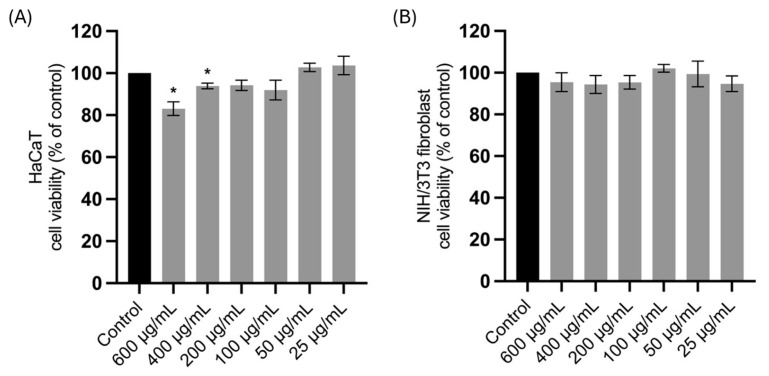
Effect of the aqueous leaf extract of *L. rectus* (LRAE) on cell viability of (**A**) HaCaT keratinocytes and (**B**) NIH/3T3 fibroblasts through resazurin assay. The results are expressed as a percentage of cell viability compared to the control. Columns and bars represent mean and standard deviation, respectively (*n* = 3 independent experiments performed in duplicate). Statistical analysis was performed by one-way ANOVA followed by Dunnett’s Multiple Comparison Test: * *p* < 0.05, compared to the control.

The resazurin-based metabolic assay was used to evaluate the effect of LRAE on the viability of HaCaT keratinocytes (Figure 1A) and NIH/3T3 fibroblasts (Figure 1B). The results indicate that LRAE is safe for these cell lines, maintaining cell viability above 80%. This threshold is commonly interpreted as non-cytotoxic and is also considered as such according to the ISO 10993-5:2009 standard [24].

### 2.3. Wound-Healing Activity of the Aqueous Leaf Extract of L. rectus

**Figure 2:**
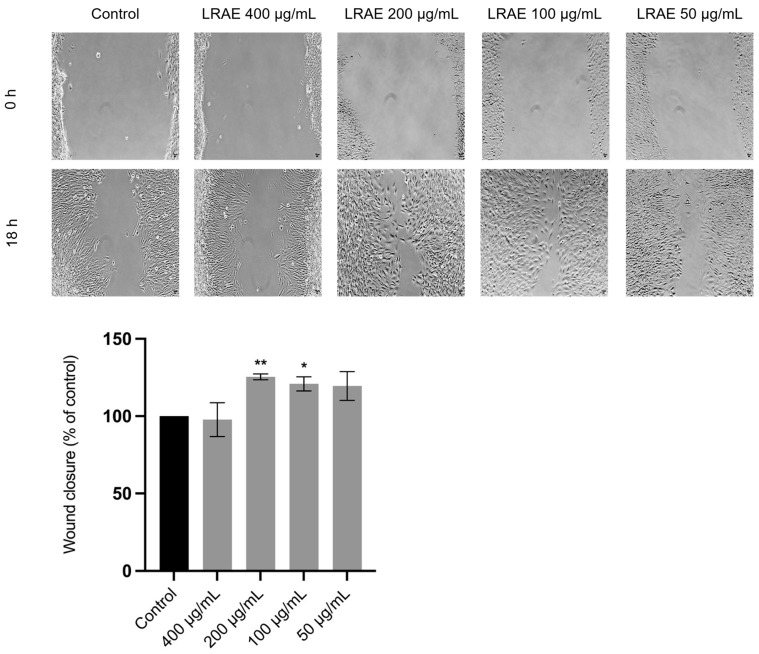
Evaluation of the wound healing effect of the aqueous leaf extract of *Lotus rectus* L. (LRAE) on NIH/3T3 fibroblasts (Scratch assay). Data are expressed as a comparison, as a percentage of wound closure relative to control, between the wound area at 0 and 18 hours. Columns and bars represent mean and standard deviation, respectively (*n* = 3 independent experiments performed in duplicate). Statistical analysis was performed by one-way ANOVA followed by Dunnett’s Multiple Comparison Test: * *p* < 0.05, ** *p* < 0.01, compared to control. Magnification = 10×; scale bar = 10 µm.

The scratch assay was employed to assess the wound-healing potential of LRAE in NIH/3T3 fibroblasts (Figure 2). This method allows the evaluation of cell migration and wound closure by monitoring the ability of cells to repopulate an artificially created cell-free area in a confluent monolayer. In this context, to ensure that the observed effects are primarily due to enhanced cell migration rather than increased cell proliferation, strategies such as reducing fetal bovine serum (FBS) concentration from 10% to 2% were employed. Nevertheless, a background contribution from cell proliferation cannot be completely excluded, and therefore, the results should be interpreted with caution [25]. Treatment with LRAE for 18 h at concentrations of 100 and 200 µg/mL significantly increased the fibroblast migration rate by more than 20% compared with the untreated control group (*p* < 0.05 and *p* < 0.01, respectively). These preliminary results suggest that LRAE may enhance scratch closure under the experimental conditions tested in vitro.

### 2.4. Evaluation of the Enzyme-Inhibitory Activity of the Aqueous Leaf Extract of L. rectus L.

The enzymatic inhibitory activity of LRAE was evaluated against several enzymes associated with oxidative stress, skin pigmentation, and extracellular matrix remodeling (Table 3). In this context, LRAE reached 50% enzymatic inhibition for xanthine oxidase (IC_50_ = 93.84 ± 4.44 µg/mL) and tyrosinase (IC_50_ = 581.01 ± 16.32 µg/mL), with these effects occurring at concentrations within the range considered biologically safe for the evaluated cell lines. In this regard, notable differences can be observed between both enzymes, with xanthine oxidase showing a much more pronounced inhibition than tyrosinase, which is inhibited to a more modest extent. Regarding collagenase inhibition, 50% inhibition was not achieved at 600 µg/mL, the highest concentration tested. Accordingly, the results are presented as percentage inhibition at the evaluated doses. At 600 µg/mL, LRAE inhibited collagenase activity by 48.93 ± 8.96%, while at 300 µg/mL an inhibition of 22.26 ± 3.46% was observed, indicating a dose-dependent effect within the tested range. Although the inhibitory effect observed at 600 µg/mL was lower than that of the reference compound epigallocatechin gallate (EGCG), which showed an inhibition of 61.39 ± 4.04% at 200 µg/mL, no significant differences were found between both groups (*p* > 0.05). In the case of the standard, a dose-dependent inhibitory effect was also observed, with an inhibition of 37.74 ± 9.49% at 100 µg/mL. Interestingly, the inhibitory effect of LRAE against collagenase appeared to be selective among the ECM-degrading enzymes evaluated, as its activity against hyaluronidase was minimal (4.05 ± 1.10%) even at 600 µg/mL.

### 2.5. Antioxidant Capacity of the Aqueous Leaf Extract of L. rectus L. in Cell-Free Systems

**Figure 3:**
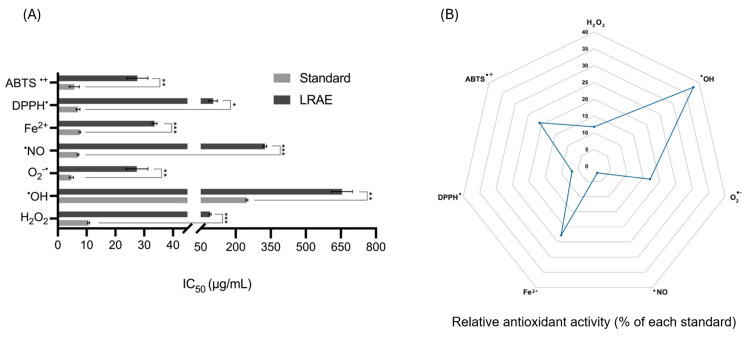
Inhibitory activity of the aqueous leaf extract of *Lotus rectus* L. (LRAE) against selected biological and synthetic pro-oxidant systems: (**A**) Half-maximal inhibitory concentrations (IC50, µg/mL) of LRAE compared with the corresponding standard compounds. (**B**) Relative antioxidant activity profile of LRAE expressed as percentage of the reference standard activity calculated as (IC50 standard/IC50 sample) × 100. Data are expressed as mean ± standard deviation (*n* = 3 independent experiments performed in duplicate). Statistical analysis was performed either by Student’s *t*-test for independent samples with equal variances or Welch’s *t*-test for samples with unequal variances: * *p* < 0.05, ** *p* < 0.01, *** *p* < 0.001.

The antioxidant activity of LRAE against different biological and synthetic pro-oxidants is summarized in Figure 3. As shown in panel A, the lowest IC_50_ values were observed for superoxide anion (O_2_^•−^) (27.45 ± 3.81 µg/mL), ABTS^•+^ radical cation (27.62 ± 3.64 µg/mL) and ferrous iron (Fe^2+^) (33.60 ± 0.83 µg/mL), indicating a comparatively higher inhibitory potency toward these systems. Moderate activity was observed against hydrogen peroxide (H_2_O_2_) (90.44 ± 4.14 µg/mL). In contrast, weaker inhibition was detected for DPPH^•^ (103.55 ± 18.07 µg/mL) and particularly for nitric oxide (^•^NO) (325.26 ± 7.13 µg/mL). The highest IC_50_ value corresponded to hydroxyl radicals (^•^OH) (655.09 ± 44.29 µg/mL), indicating the lowest inhibitory potency among the evaluated systems. However, to provide a more comprehensive comparison in absolute and relative terms, panel B depicts the relative antioxidant activity profile of LRAE, expressed as the percentage of the activity of the corresponding standard. This representation highlights the relative performance of the extract across the different oxidant systems, showing higher relative activities against ^•^OH, Fe^2+^, ABTS^•+^, and O_2_^•−^, while lower relative activities were observed for H_2_O_2_, DPPH^•^, and particularly ^•^NO. Together, both representations provide complementary information, illustrating the system-dependent inhibitory behavior of LRAE against a range of biologically relevant oxidant species and synthetic radical systems.

### 2.6. Assessment of the Antimycotic Potential of the Aqueous Leaf Extract of L. rectus L. Against Dermatophytes and Yeasts

The antifungal activity of LRAE against dermatophytes and yeast strains is summarized in Table 4. The extract exhibited limited fungistatic and fungicidal activity only at relatively high concentrations against the dermatophyte *Epidermophyton floccosum* FF9 and *Microsporum canis* FF1, both isolated from clinical samples of nail and skin. In these cases, LRAE showed minimum inhibitory concentration (MIC) and minimum lethal concentration (MLC) values of 1000 µg/mL for *E. floccosum* and 800 µg/mL for *M. canis*. For the remaining dermatophytes evaluated, as well as for all tested *Candida* species, the extract did not exhibit inhibitory activity within the concentration range tested (MIC > 1000 µg/mL). MIC values of the reference antifungal agent fluconazole confirm the susceptibility of the fungal panel under our experimental conditions.

## 3. Discussion

*Lotus rectus* L. (Fabaceae) is a perennial herbaceous species native to the Mediterranean basin that typically grows along watercourse margins and in permanently moist soils across a wide range of substrates, from sea level up to approximately 1300 m above sea level. In the Iberian Peninsula, it has been traditionally used in folk medicine as a vulnerary remedy for the treatment of wounds, burns, and ulcers. It has also been employed in ethnoveterinary practices to treat animal wounds and external microbial and parasitic infections [11,12,13]. Beyond its traditional medicinal use, this species has attracted attention as an agronomic temperate forage legume, due to its ease of cultivation and management in semi-arid environments, as well as its reported anthelmintic activity against intestinal parasites in sheep [16,26,27]. However, despite its well-documented traditional use and its ecological and agricultural relevance, the bioactive potential of *L. rectus* in relation to wound healing and skin health remains largely unexplored.

Previous phytochemical studies of species belonging to the genus *Lotus* have shown that these plants are particularly rich in phenolic constituents, mainly flavonoids and proanthocyanidins, including derivatives of kaempferol, quercetin, and catechin-type flavan-3-ols [19]. Specifically, in the case of *L. rectus*, the limited phytochemical studies available have reported the occurrence of kaempferol 3,7-dirhamnoside-type flavonoids together with condensed tannins composed of mixed prodelphinidin and procyanidin polymers [28,29]. Consistent with this characteristic phytochemical profile, the analysis of the aqueous leaf extract of *L. rectus* L. (LRAE) revealed high relative levels of total flavonoids and tannins, the latter being mainly represented by proanthocyanidins (Table 1). In line with this, the limited presence of hydroxycinnamic acids in LRAE appears consistent with previous reports on aqueous extracts from other species within the same genus [30]. Additionally, several phenolic acids and flavonoid derivatives commonly reported in *Lotus* species were detected (Table 2). Specifically, considerable levels of kaempferol-3,7-O-α-di-rhamnopyranoside were detected (approximately 6 mg/g of dry extract), a flavonol diglycoside commonly found in other *Lotus* species [31].

Wound healing is a multifactorial process involving a complex interplay of biochemical and cellular events, including cell migration, extracellular matrix (ECM) remodeling and oxidative balance regulation. Indeed, numerous studies have highlighted that secondary metabolites from the plant kingdom, such as phenolic compounds, may contribute to the healing of both acute and chronic wounds through multiple mechanisms. Consequently, plant-derived extracts traditionally associated with wound treatment are often investigated through a combination of complementary bioassays that target different mechanisms involved in tissue repair [32]. In this context LRAE proved to be cytocompatible with HaCaT keratinocytes and NIH/3T3 fibroblasts and was associated with an increase of up to 20% in fibroblast migration (*p* < 0.01) at concentrations that did not compromise cell viability in vitro (Figure 2). Given the pivotal role of fibroblasts in wound healing, these results provide preliminary in vitro evidence of bioactivity in relation to the traditional use of this species, highlighting its potential interest for further investigation [33]. These findings may be partly attributed to the high content of flavonols and proanthocyanidins in the extract. In this regard, different kaempferol glycosides have been shown to promote cell migration in human dermal fibroblasts in vitro, whereas quercetin treatment appears to increase surface αV integrin levels in L929 fibroblasts, which is associated with enhanced cell migration [34,35]. Overall, in vitro and in vivo studies suggest that flavonoids may enhance fibroblast and keratinocyte migration through modulation of signaling pathways involved in cell migration, including MAPK pathways (ERK, p38, and JNK) and the PI3K–Akt pathway [36]. Moreover, together with tannins, these compounds appear to upregulate the production of growth factors involved in wound-related cell migration, such as FGF, TGF-β, and VEGF [36,37]. To the best of our knowledge, evidence within the genus *Lotus* regarding effects on cell migration in the context of wound healing is very limited and largely restricted to studies on cancer cell migration [31], most of which focus on lectins isolated from these species [38].

In addition to the effects observed on fibroblast behavior, LRAE also demonstrated antioxidant activity, including the inhibition of xanthine oxidase, a molybdoflavin enzyme involved in purine catabolism and reactive oxygen species (ROS) generation in wounded tissues [39,40]. ROS play a dual role in wound healing, as moderate levels are required for antimicrobial defense and cell signaling, whereas excessive and persistent oxidative stress contributes to impaired cellular proliferation, sustained inflammation, and wound chronicity [39]. Notably, fibroblasts derived from chronic wounds have been reported to produce higher levels of ROS than normal dermal fibroblasts [41]. In this context, the ability of LRAE to modulate different ROS, together with its inhibitory effect on xanthine oxidase and its capacity to chelate ferrous ion (Fe^2+^), suggests a potential involvement in the modulation of oxidative processes, although these findings still require confirmation in biological systems. Since xanthine oxidase is a relevant source of hydrogen peroxide (H_2_O_2_) and superoxide anion (O_2_^•−^) during tissue injury, and Fe^2+^ participates in the generation of highly reactive hydroxyl radicals (^•^OH) through Fenton-type reactions, the observed effects may reflect a complementary mechanism involving both direct ROS scavenging and reduced ROS formation [39,40,42]. Although the scavenging of ^•^OH was only observed at relatively high concentrations, this activity remains noteworthy given their high reactivity and damaging potential, particularly considering the favorable IC_50_ ratio relative to the standard ascorbic acid. In relation to aqueous extracts from *Lotus* species, other studies have indicated that flavonoids are primarily responsible for their overall antioxidant capacity [43], with polar extracts often exhibiting higher antioxidant activity than less polar fractions [30,31]. Beyond ROS, the relatively low nitric oxide (^•^NO) scavenging activity exhibited by the extract is consistent with that reported for other species within the same genus, where flavonol aglycones appear to exert a greater effect than their glycosylated derivatives [44].

Moreover, regarding the xanthine oxidase inhibition by LRAE, this activity may be partly attributed to the presence of polyphenols in the extract, particularly flavonols such as quercetin and kaempferol, which are among the most extensively studied natural inhibitors of this enzyme and appear to act through a non-competitive mechanism [45,46]. These compounds may inhibit xanthine oxidase primarily through hydrogen bonding interactions and active-site occupation, often involving additional interactions with the flavin adenine dinucleotide (FAD) domain and a mixed-type inhibition mechanism that can hinder substrate access and superoxide anion diffusion [45]. A planar structure and the number and position of hydroxyl groups are key for effective binding, with a catechol moiety in the B-ring enhancing activity and explaining the higher potency of quercetin compared to kaempferol. However, an excess of hydroxyl groups, as in glycosylated derivatives, generally reduces inhibitory activity due to steric hindrance at the catalytic site of the enzyme [47]. In addition, non-planar flavan-3-ols and condensed tannins, such as catechins and proanthocyanidins, may also contribute significantly through alternative mechanisms, including direct protein binding and the formation of enzyme–polyphenol complexes stabilized by hydrophobic interactions [48]. In relation to other species of the genus *Lotus*, the half maximum inhibitory concentration obtained in the present study (IC_50_ ≈ 94 µg/mL) is consistent with the range reported for flavonol glycosides-rich extracts from other *Lotus* species (IC_50_ = 55–260 µg/mL) [46]. Collectively, these preliminary results illustrate the antioxidant potential of LRAE and justify further studies to better understand the biological relevance of this activity [42]. In this context, examples of the use of *Lotus* species as cosmetic ingredients already exist, such as the aqueous leaf extract of *L. maritimus*, which has been incorporated into a topical anti-cellulite gel marketed in Canada due to its antioxidant properties [22].

In addition, LRAE demonstrated moderate inhibitory activity against collagenase and tyrosinase, although to a lesser extent than for xanthine oxidase (Table 3). Collagenase is a matrix metalloproteinase (zinc-dependent endopeptidase) that specifically cleaves collagen, the main structural component of the cutaneous ECM. During normal wound healing, its activity is transiently elevated in the inflammatory and early proliferative phases and subsequently decreases during tissue contraction and remodeling, remaining under tight control of endogenous inhibitors [49,50]. However, in chronic wounds, this protease–antiprotease balance shifts toward excessive proteolytic activity, resulting in sustained ECM degradation and impaired tissue repair [51]. In this context, although LRAE exhibited moderate inhibition of collagenase activity (~50%) at 600 μg/mL, and minimal inhibition of hyaluronidase, these findings provide a preliminary basis for future studies aimed at identifying the constituents responsible for this activity, as related evidence within the genus *Lotus* remains scarce. Among the secondary metabolites present in LRAE, flavonols such as kaempferol and quercetin and their glycosides, flavan-3-ols such as gallocatechin, as well as condensed tannins, have been frequently associated with collagenase inhibition [52]. This activity appears to be related to the presence of a C-3 hydroxyl group in flavonols, catechol-type substitution patterns, and the high density and specific arrangement of hydroxyl groups, which enable both coordination to the catalytic Zn^2+^ ion and the formation of multiple hydrogen bonds with surrounding residues. Notably, glycosylated flavonols can further enhance these interactions by providing additional hydrogen-bonding interactions within the enzyme active site [53]. Moreover, LRAE showed modest inhibitory activity in the tyrosinase inhibition assay (Table 3), a copper-containing oxidoreductase involved in melanin biosynthesis [54]. Due to the pathophysiological implications of this enzyme, the search for tyrosinase inhibitors has relevance for the development of skin-depigmenting agents as well as for therapeutic strategies targeting melanogenesis-related disorders such as melanoma or Parkinson’s disease [55]. In this context, the modest inhibitory activity against tyrosinase exhibited by LRAE appears to be consistent with previous reports on polar extracts from other *Lotus* species rich in flavonol glycosides, which have shown limited or no activity against this enzyme. In fact, increased inhibition observed in less polar extracts suggests that compounds other than the major glycosylated flavonoids may contribute to this effect [30,56]. Such relatively modest inhibitory effects may also reflect apparent inhibitory activity rather than true enzyme inhibition. This consideration is particularly relevant given the multiple ways in which phenolic compounds may interfere with tyrosinase assays, including their role as reducing agents, alternative substrates, or quinone scavengers [57]. Therefore, further studies are needed to determine the effect of less polar fractions or extracts of *L. rectus* and whether these preliminary findings reflect the presence of true tyrosinase inhibitors or compounds that act through indirect mechanisms.

Finally, the antifungal activity of LRAE was evaluated against dermatophytes and *Candida* species. These fungi are responsible for common skin infections affecting millions of people worldwide and represent the most prevalent skin disease in Europe, contributing to a substantial economic burden [58,59]. LRAE only showed minimal antifungal activity against the dermatophytes *Epidermophyton floccosum* and *Microsporum canis* under our experimental conditions. Although this activity was observed only at relatively high concentrations, these results represent the first report of antifungal activity from a *Lotus* species against dermatophytes. Although less polar extracts from other *Lotus* species have previously shown moderate activity against *C. albicans* [60,61], most aqueous or hydroalcoholic extracts have not demonstrated notable anti-*Candida* activity [61,62,63], with the exception of the aqueous extract of *L. lalambensis*, whose content of the coumarin derivative 5′-hydroxy auraptene appears to be associated with its moderate antifungal activity [64]. Flavonoids present in LRAE, such as chrysin, pinocembrin, and flavonol glycosides, have shown low to moderate antidermatophytic activity. Tannins, namely phlorotannins and ellagitannins, have also been reported to exhibit antidermatophytic activity, although the available evidence remains limited [65].

## 4. Materials and Methods

### 4.1. Reagents and Standards

All solvents used for extraction and chromatographic analyses were obtained from VWR International (Rosny-sous-Bois, France). Phenolic reference standards were supplied by Extrasynthese (Genay, France) and Merck KGaA (Darmstadt, Germany). Kaempferol diglycosides were kindly provided by Prof. C. Martín-Cordero (Faculty of Pharmacy, University of Seville, Seville, Spain). Most reagents, including those used in the quantitative phytochemical analysis, were purchased from Sigma-Aldrich (St. Louis, MO, USA), except for the Folin–Ciocalteu reagent, which was acquired from Panreac AppliChem (Monza, Italy), 1-vinyl-2-pyrrolidinone polymer (PVPP), which was purchased from Indagoochem (Barcelona, Spain), and the reagents used in the free radical scavenging assays and enzymatic assays, which were mainly obtained from TCI Europe N.V. (Zwijndrecht, Belgium).

### 4.2. Plant Material and Aqueous Extract Preparation

The aerial parts of *Lotus rectus* L. were collected in early July 2023 in the northern region of the province of Huelva (southwestern Spain), at GPS coordinates 37°52′27.2″ N 6°46′54.6″ W, at 595 m a.s.l. The collection of plant material was authorized by the competent regional authority (Ref.: AJLF/ifs; Expte. 1 17 2-2022-1). Botanical identification was carried out by Prof. P. García-Murillo (Faculty of Pharmacy, University of Seville, Seville, Spain), and a voucher specimen was deposited in the Herbarium of the University of Seville (voucher specimen number: SEV289986). After collection, the plant material was rinsed with distilled water and air-dried in the shade. Once dried, the leaves were separated from the stems and ground to a particle size of 5–10 mm. The dried leaves were then extracted with distilled water (30 g/L) at boiling temperature for 5 min in order to reproduce a traditional decoction process of the plant. The mixture was subsequently filtered through Whatman^®^ Grade 1 paper, frozen, and lyophilized.

### 4.3. Determination of Major Phenolic Constituents

The major phenolic constituents were quantified using classical spectrophotometric assays adapted to a 96-well microplate format, following established methodologies reported in the literature and previously applied in related studies [66,67]. All experimental data, except total tannin content, were expressed as milligrams of standard equivalents per gram of dry extract.

Briefly, total phenolic content was determined using a Folin–Ciocalteu assay modified for microplate analysis, measuring optical density (OD) at 750 nm (OD_750_) and using gallic acid as the external standard [68,69]. Flavonoid content was estimated using the aluminum chloride complexation method described by Lamaison and Carnat [70], with rutin as the external standard (OD_405_). Hydroxycinnamic acid derivatives were quantified according to the method described by Arnow [71], adapted for microplate analysis [72]. Caffeic acid was used as the reference standard, and OD was measured at 490 nm.

Total tannin content was determined using the method described by Makkar [73], adapted for microplate reading according to Nickerson et al. [74]. Tannic acid served as the reference standard (OD_750_). The results were expressed as % (*w*/*w*) of tannin-related phenols relative to the total phenolic content (TRP) determined by the Folin–Ciocalteu assay. To further characterize the predominant tannin type, proanthocyanidin content in the extract was quantified using the vanillin assay according to Sun et al. [75], with modifications for a multi-well plate format [76]. Catechin was used as the reference compound (OD_490_). All absorbance measurements were performed using an iMark™ microplate absorbance reader (Bio-Rad Laboratories, Inc., Hercules, CA, USA).

### 4.4. Identification and Quantification of Phenolic Compounds by UHPLC-HRMS/MS

Phytochemical characterization was performed by ultra-high-performance liquid chromatography coupled to high-resolution tandem mass spectrometry (UHPLC-HRMS/MS) using a Dionex Ultimate 3000 RS UHPLC system (Thermo Fisher Scientific, San Jose, CA, USA) coupled to a Q-Exactive Orbitrap high-resolution mass spectrometer equipped with a heated electrospray ionization source (HESI-II). Analyses were conducted in negative ionization mode. Instrument control and data acquisition were carried out using Xcalibur v4.3 (Thermo Fisher Scientific, San Jose, CA, USA). Chromatographic separation was achieved on an Acquity BEH C18 reversed-phase column (2.1 × 100 mm, 1.7 µm; Waters, Milford, MA, USA). The mobile phase consisted of solvent A (0.1% formic acid in water) and solvent B (0.1% formic acid in methanol). Elution was performed using a gradient program as follows: 0–1 min, 5% B (isocratic); 1–10 min, linear increase to 100% B; 10–12 min, 100% B (isocratic); and 12–15 min, re-equilibration to the initial conditions (5% B). The flow rate was set at 0.5 mL min^−1^, and the injection volume was 5 µL. Nitrogen was used as sheath, auxiliary, and sweep gas at flow rates of 60, 25, and 0 arbitrary units, respectively. The HESI-II heater temperature was set to 400 °C, the capillary temperature to 320 °C, and the spray voltage to −3.0 kV. The S-Lens RF level was set to 50 V. Full-scan MS spectra were acquired over an *m*/*z* range of 50–750 with a resolving power of 70,000 FWHM (at *m*/*z* 200). The automatic gain control (AGC) target was set to 3.0 × 10^6^, with a maximum injection time of 200 ms. Data-dependent MS/MS acquisition was subsequently performed in Top5 mode, applying stepped normalized collision energies of 30, 60, and 90 eV. Fragment ion spectra were recorded at a resolution of 17,500 FWHM (*m*/*z* 200), with an AGC target of 2 × 10^5^ and a maximum injection time of 50 ms. An isolation window of 0.7 *m*/*z* was used, and MS/MS acquisition was triggered at an intensity threshold of 1.6 × 10^5^ [67,77].

Compound identification was performed with Trace Finder v 5.1 software (Thermo Fisher Scientific, San Jose, CA, USA). Identification was achieved by comparing retention times, the exact masses of the pseudomolecular ions ([M-H]^−^), and their fragment ions (with a maximum deviation of 5 ppm) against an internal database of phenolic standard compounds provided by the Centro de Investigación y Transferencia of the University of Seville (CITIUS). Isotopic pattern match scores greater than 80% were required for confirmation.

Compound quantification was carried out using an external calibration approach based on commercially available reference standards, under the same instrumental and chromatographic conditions described above for the identification of phenolic compounds. Calibration curves were constructed by injecting standard solutions (0.01–10 µg/mL) of kaempferol-3,7-di-O-rhamnoside (y = 1.269 × 10^5^x  + 6.417 × 10^7^; R^2^ = 0.995), hyperoside (y = 1.778 × 10^5^x + 8.738 × 10^5^; R^2^ = 0.997), caffeic acid (y = 5.578 × 10^5^x − 2.677 × 10^6^; R^2^ = 0.998) and naringenin (y = 1.055 × 10^6^x − 7.949 × 10^6^; R^2^ = 0.996). The limits of detection (LOD) and limits of quantification (LOQ) were determined according to Uhrovčík [78], and the linear response of the method was verified across the selected calibration range (Table 5). Only compounds with concentrations above the LOQ were considered for quantitative analysis. The results were expressed as milligrams of each identified compound per gram of dry extract, reported as mean ± standard deviation (SD) of three independent experiments.

### 4.5. Cell Culture and Cell Viability

Human keratinocytes (HaCaT cell line, CLS Cell Lines Service GmbH, Cytion, Eppelheim, Germany), originally described by Boukamp et al. [79], and murine fibroblasts (NIH/3T3, ATCC CRL-1658, Manassas, VA, USA) were maintained in Dulbecco’s Modified Eagle Medium (DMEM; 41965-039, Gibco, Thermo Fisher Scientific Inc., Waltham, MA, USA) supplemented with 10% (*v*/*v*) heat-inactivated fetal bovine serum (FBS; A5256701, Gibco) and 1% (*v*/*v*) penicillin–streptomycin (15070-063, Gibco). Cell culture conditions followed protocols previously established [67,80]. Prof. José Calderón Montaño (Faculty of Pharmacy, University of Seville) supervised the handling of human keratinocyte cultures.

Cell viability following treatment with the aqueous leaf extract of *L. rectus* L. (LRAE) was assessed using the resazurin sodium salt reduction assay (Alamar Blue), as previously described in related studies [80,81]. HaCaT keratinocytes (2 × 10^5^ cells/mL) and NIH/3T3 fibroblasts (5 × 10^4^ cells/mL) were seeded into 96-well plates (HaCaT) or 48-well plates (NIH/3T3) and allowed to adhere overnight. Cells were then exposed for 24 h to different concentrations of LRAE (25–600 µg/mL). After the treatment period, the culture medium was removed, and the cells were gently rinsed with phosphate-buffered saline (PBS). A 500 µM resazurin solution prepared in DMEM (1:10) was then added to each well. Plates were incubated for 2 h at 37 °C in a humidified atmosphere containing 5% CO_2_, after which optical density was recorded at 570 nm using 620 nm as the reference wavelength with an Infinite^®^ microplate reader (Tecan Austria GmbH, Salzburg, Austria). Cell viability was expressed as the percentage relative to untreated control cells for each cell line.

### 4.6. Scratch Assay

The effect of LRAE on fibroblast migratory capacity was evaluated using a wound-healing (scratch) assay following the methodology described by Martinotti and Ranzato [25] with minor adaptations previously implemented [80]. Briefly, NIH/3T3 fibroblasts were seeded in 12-well plates at a density of 3 × 10^5^ cells per well and allowed to grow for 24 h to form a confluent monolayer. A straight scratch was then created across the cell layer using a sterile 20 µL pipette tip to generate a cell-free gap. Detached cells were removed by rinsing once with sterile phosphate-buffered saline (PBS). The remaining adherent cells were subsequently maintained in culture medium containing 2% FBS, either in the absence (control) or presence of LRAE at concentrations ranging from 50 to 400 µg/mL. This marked reduction in FBS concentration is intended to decrease the rate of cell proliferation in order to assess the effects of the treatment on cell migration. Images of the cell-free area were acquired by phase-contrast microscopy (Carl Zeiss, Oberkochen, Germany) at 10× immediately after scratch formation (0 h) and after 18 h of incubation. The original representative images used in Figure 2 of this article are available in the Supplementary Materials (Supplementary Document S2). The wounded area in each micrograph was quantified using an ImageJ2 v.2.16/Fiji plugin, following the procedure described by Suarez-Arnedo et al. [82]. The percentage of wound closure was determined by comparing treated samples with the untreated control condition, with the control defined as the reference value (100%) and the results expressed relative to this baseline.

### 4.7. Enzyme-Inhibitory Assays

#### 4.7.1. Xanthine Oxidase

The inhibitory activity of LRAE against xanthine oxidase (EC 1.17.3.2, from bovine milk, Sigma-Aldrich) was evaluated by monitoring uric acid formation at 295 nm following the procedure described by Nile et al. [83] with minor modifications previously detailed [67]. Allopurinol was included as a positive reference inhibitor. Briefly, 100 µL of xanthine oxidase solution (0.8 U/mL) was combined with 10 µL of LRAE or allopurinol and incubated for 15 min at room temperature. The reaction was then initiated by adding phosphate buffer (50 mM, pH 7.4), EDTA (10 mM), and xanthine (10 mM) as substrate. The increase in absorbance was monitored at 295 nm for 2 min using a UV–VIS spectrophotometer (UV-1800, Shimadzu Corporation, Kyoto, Japan). The results were expressed as the half-maximal inhibitory concentration (IC_50_, µg/mL).

#### 4.7.2. Collagenase

The collagenase inhibitory activity of LRAE was investigated using an Azocoll^TM^-based colorimetric assay according to Wang et al. [84] with slight adaptations [85]. In brief, collagenase (EC 3.4.24.3, from *Clostridium histolyticum*, 200 U/mL, Sigma-Aldrich) was prepared in 0.1 M Tris–HCl buffer (pH 7.0) and incubated with Azocoll^TM^-impregnated collagen in the presence of LRAE or the reference inhibitor epigallocatechin gallate (EGCG, 200 µg/mL) in Eppendorf tubes. Due to the technically demanding nature of the assay, the inhibitory effect at the highest concentration, confirmed to be non-cytotoxic for the cell lines (600 µg/mL), was used as an initial reference point to assess collagenase inhibition. An additional lower dose (300 µg/mL) was evaluated to examine dose-dependent variability. Regarding EGCG, concentrations with established inhibitory effects (200 and 100 µg/mL) were evaluated for the same purpose. The mixtures were maintained at 43 °C for 1 h to allow enzymatic degradation of the dyed collagen substrate. After incubation, samples were centrifuged at 3000 rpm for 10 min, and the absorbance of the resulting supernatants was measured at 550 nm using a Multiskan™ FC microplate reader (Thermo Fisher Scientific Inc., Waltham, MA, USA). The inhibitory effect was expressed as the percentage of collagenase inhibition relative to the untreated control.

#### 4.7.3. Hyaluronidase

The inhibitory activity of LRAE against hyaluronidase was assessed following the procedure stated by Sahasrabud and Deodhar [86], with adaptations reported by Jiratchayamaethasakul et al. [85]. Briefly, LRAE solutions (up to 600 µg/mL), or the reference compound tannic acid, were prepared in 0.1 M acetate buffer (pH 3.6) and combined with hyaluronidase (EC 3.2.1.35, 7900 U/mL, from bovine testis, Sigma-Aldrich) in Eppendorf tubes. The enzymatic reaction was subsequently carried out following the reported protocols through the sequential addition of calcium chloride (12.5 mM) and hyaluronic acid (1.2 mg/mL) as substrate. The reaction was then terminated using potassium tetraborate (0.2 M) and sodium hydroxide (0.4 M), and color development was achieved with 4-(dimethylamino)benzaldehyde (DMAB) solution. Absorbance was recorded at 585 nm. The inhibitory activity was expressed as the IC_50_ (µg/mL).

#### 4.7.4. Tyrosinase

The tyrosinase inhibitory activity of LRAE was evaluated using the method described by No et al. [87]. Briefly, a reaction mixture was prepared in 96-well microplates containing 0.1 M phosphate-buffered saline (PBS, pH 6.8), LRAE up to 600 µg/mL or the reference inhibitor kojic acid, tyrosinase (EC 1.14.18.1, 1500 U/mL, from mushroom, Sigma-Aldrich), and L-tyrosine (1.5 mM) as substrate. After an incubation period of 15 min, the dopachrome formation was monitored by measuring absorbance at 490 nm. The inhibitory activity was expressed as the IC_50_ (µg/mL).

### 4.8. Determination of the Antioxidant Activity of LRAE

#### 4.8.1. Reactive Oxygen Species, Nitric Oxide and Transition Metals (Biological Pro-Oxidants)

Assays targeting biologically and synthetically relevant pro-oxidant systems were performed as previously described in our earlier work [67]. Specifically, the ROS-scavenging activity of LRAE was evaluated against the hydroxyl radicals (OH^•^), the superoxide anion (O_2_^•−^) and hydrogen peroxide (H_2_O_2_), while activity against reactive nitrogen species (RNS) was assessed using nitric oxide (^•^NO) as the target species. The transition metal chelating activity was measured against the ferrous ion (Fe^2+^).

Scavenging capacity towards OH^•^ radicals was assessed through the salicylate assay described by Guo et al. [88], using ascorbic acid as the reference antioxidant and incorporating modifications suitable for microplate-based analysis [67]. Briefly, 30 µL of sodium salicylate (20 mM), 100 µL of ferrous sulfate (1.5 mM), 50 µL of the sample and 70 µL of H_2_O_2_ (6 mM) were mixed in a 96-well plate. After 1 h at 37 °C the absorbance was measured at 540 nm.

The activity against O_2_^•−^ was evaluated using the hypoxanthine–xanthine oxidase coupled system reported by Aruoma et al. [89], with volumes adjusted to a microplate format and gallic acid used as the standard compound. Briefly, 62 µL of sodium phosphate buffer (50 mM), 10 µL of ethylenediaminetetraacetic acid disodium (EDTA-Na_2_) (15 mM), 15 µL of hypoxanthine (3 mM), 25 µL of nitroblue tetrazoyl (NBT) (0.6 mM), 12 µL of the sample and 25 µL of xanthine oxidase (0.36 U/mL) were mixed in a microtiter plate. Immediately after the addition of all reagents, the absorbance was measured at 560 nm every five minutes for 40 min.

The ability of the extract to neutralize H_2_O_2_ was determined following the method of Aruoma et al. [90] with minor adjustments introduced by García-Prado [91]. Briefly, 235 µL of sodium phosphate buffer (0.01 M), 3 µL of the sample and 26 µL of H_2_O_2_ (1 mM) were mixed in a microtiter plate. After 30 min, 13 µL of guaiacol (0.2% *v*/*v*) and 3 µL of horseradish peroxidase (900 U/mL) were added to the mixture. After 10 min the absorbance was measured at 450 nm. Trolox was used as the reference standard.

The ^•^NO scavenging activity of LRAE was evaluated using the Griess reaction with sodium nitroprusside (SNP) as the radical source [92]. Briefly, 20 µL of the sample was added to 1 mL of SNP (5 mM in phosphate buffer) and incubated at 37 °C for 150 min. After this period, equal volumes of this solution and Griess reagent (0.1% (*w*/*v*) N-(1-naphthyl) ethylenediamine dihydrochloride and 1% (*w*/*v*) sulfanilamide prepared in 5% (*w*/*v*) H_3_PO_4_) were mixed and the absorbance was registered at 550 nm. Caffeic acid was used as a reference standard.

Finally, the ferrous ion chelating ability of the extract was determined using the method originally described by Carter [93], adapted for microplate-based measurements following the modifications reported by Santos et al. [94]. This method is based on the formation of a Fe^2+^–ferrozine chromogenic complex, produced using ferrous sulphate as the Fe^2+^ donor. Briefly, 50 µL of the sample was mixed with 160 µL of distilled water and 20 µL of ferrous sulphate heptahydrate (85 µg/mL) in a microtiter plate. After 5 min, 30 µL of ferrozine (400 µg/mL) was added to this solution, and after 15 min, the absorbance was measured at 540 nm. Disodium ethylenediaminetetraacetate (EDTA-Na_2_) was used as the reference metal chelator.

In all cases, the antioxidant effectiveness was expressed as the half-maximal inhibitory concentration (IC_50_, µg/mL). The relative antioxidant activity of LRAE was additionally expressed as the percentage of activity relative to the corresponding reference compound, calculated as (IC_50_ standard/IC_50_ sample) × 100.

#### 4.8.2. Non-Biological Radicals

The radical scavenging capacity of LRAE was examined using two widely employed synthetic radical systems, DPPH^•^ and ABTS^•+^, corresponding to 1,1-diphenyl-2-picrylhydrazyl and 2,2′-azino-bis(3-ethylbenzothiazoline-6-sulfonic acid) diammonium salt, respectively [66]. For the DPPH^•^ assay, the protocol originally proposed by Sánchez-Moreno et al. [95] was applied, incorporating modifications that allow microplate-based measurements as described by Cuendet et al. [96]. Briefly, 100 µL of the sample was added to a 96-well microtiter plate, followed by the addition of 100 µL ethanol and 50 µL of DPPH solution (0.022% *w*/*v* in ethanol). The plate was incubated under agitation for 30 min in the dark, and the absorbance was measured at 540 nm. Trolox was used as a reference standard.

The evaluation of ABTS^•+^ radical scavenging followed the methodology of Re et al. [97], adapted to a microplate reader format [67]. Briefly, 50 µL of the sample was added to a 96-well microtiter plate followed by 200 µL of ABTS^•+^ solution (previously adjusted to an absorbance of ~0.7 at 750 nm). The plate was incubated under agitation at room temperature for 10 min in the dark, and the absorbance was measured at 750 nm. Trolox was used as the reference standard.

In both systems, the activity of the extract was expressed as the IC_50_ (µg/mL) and additionally expressed as the percentage of activity relative to the corresponding reference compound, calculated as (IC_50_ standard/IC_50_ sample) × 100.

### 4.9. Antifungal Susceptibility Testing

The antifungal activity of LRAE was investigated by determining minimum inhibitory (MIC) and minimum lethal concentrations (MLC) using the macrodilution method, following the guidelines established by the Clinical and Laboratory Standards Institute (CLSI) for yeasts (M27-A3) and filamentous fungi (M38-A2) [98,99]. Fluconazole was used as the reference antifungal agent [67]. A broad panel of fungi, including both reference strains and clinical isolates, was selected. The yeast group comprised clinical isolates associated with recurrent candidiasis (*Candida krusei* H9 and *Candida guilliermondii* MAT23) together with reference strains (*Candida albicans* ATCC 10231, *Candida parapsilosis* ATCC 90018, and *Candida tropicalis* ATCC 13803; Manassas, VA, USA). The dermatophyte panel included clinical isolates (*Epidermophyton floccosum* FF9, *Microsporum canis* FF1, and *Trichophyton mentagrophytes* FF7) obtained from nail and skin samples, as well as reference strains (*Microsporum gypseum* CECT 2908, *Trichophyton mentagrophytes* var. *interdigitale* CECT 2958, *Trichophyton rubrum* CECT 2794, and *Trichophyton verrucosum* CECT 2992; Valencia, Spain). Prior to susceptibility testing, all fungal strains were subcultured on Potato Dextrose Agar (PDA) or Sabouraud Dextrose Agar (SDA) (Oxoid, Thermo Fisher Scientific, Waltham, MA, USA) to ensure viability and purity. LRAE was tested over a concentration range of 6.25–1000 µg/mL.

### 4.10. Data Analysis

Statistical analyses were performed using GraphPad Prism version 9.5.1 (San Diego, CA, USA). The distribution of the data was first examined for normality using the Shapiro–Wilk, Kolmogorov–Smirnov, and D’Agostino–Pearson tests. For experiments involving multiple groups, comparisons were performed using one-way ANOVA followed by Dunnett’s post hoc test to evaluate differences relative to the control group. In contrast, for assays based on independent pairwise comparisons, statistical analysis was conducted using Student’s *t*-test or Welch’s *t*-test, depending on variance homogeneity, with Welch’s correction applied when equal variances could not be assumed. The choice of statistical test was determined by the experimental design of each assay. Statistical significance was defined at *p* < 0.05 (*), with increasing levels of significance indicated as follows: *p* < 0.01 (**) and *p* < 0.001 (***).

## 5. Conclusions

The aqueous leaf extract of *Lotus rectus* L. (LRAE) is rich in flavonoids and proanthocyanidins, particularly flavonol glycosides, such as kaempferol-3,7-O-α-di-rhamnopyranoside. Under the experimental conditions tested in vitro, LRAE was cytocompatible with both HaCaT keratinocytes and NIH/3T3 fibroblasts across all tested concentrations and was associated with a reduction in the scratch area in vitro compared to the untreated control. Moreover, in enzymatic assays, LRAE exhibited marked xanthine oxidase inhibitory activity, whereas only moderate inhibition was observed for collagenase and tyrosinase, and no anti-hyaluronidase activity was detected. However, it displayed notable antioxidant activity against reactive oxygen species, such as hydrogen peroxide and superoxide anion, and the transition metal ferrous ion. Conversely, LRAE only showed limited antifungal effects against two clinically isolated dermatophytes, namely *Epidermophyton floccosum* and *Microsporum canis*. Overall, these findings provide preliminary in vitro evidence of bioactivity associated with the traditional use of this underexplored species in skin-related conditions. However, further studies are required to better characterize these effects, elucidate the underlying mechanisms, and determine their potential biological relevance.

## Figures and Tables

**Table 1:** Quantification of the main phenolic groups in the aqueous leaf extract of *L. rectus* (LRAE).

Assay	Content
	LRAE
Total Phenolic Content (mg GAE/g)	88.11 ± 2.28
Total Flavonoid Content (mg RE/g)	58.59 ± 2.89
Total Proanthocyanidin Content (mg CE/g)	42.54 ± 3.32
Total Tannin Content (TRP % *w*/*w*)	51.24 ± 5.37
Total Hydroxycinnamic acid Content (mg CAE/g)	ND

Data are expressed as mean ± standard deviation (*n* = 3 independent experiments performed in duplicate). Abbreviations: GAE, gallic acid equivalents; CAE, caffeic acid equivalents; RE, rutin equivalents; CE, catechin equivalents; TRP, tannin-related phenolics; ND, not detected.

**Table 2:** Phenolic compounds in the aqueous leaf extract of *L. rectus* (LRAE) analyzed by UHPLC-HRMS/MS.

No.	RT (min)	Molecular Formula	Expected *m*/*z*	Measured *m*/*z*	Error (ppm)	MS/MS Fragments	Attribution	Content mg/g Dry Extract ^1^
1	5.97	C_27_H_30_O_14_	577.1563	577.1568	0.8757	117.0344; 183.0452; 255.0301; 285.0405; 430.0905	Kaempferol-3,7-O-α-di-rhamnopyranoside	5.60 ± 0.006
2	5.67	C_21_H_20_O_12_	463.0882	463.0884	0.3222	227.0356; 243.0301; 255.0301; 271.0252; 300.0278	Hyperoside (Quercetin-3-O-galactoside)	0.03 ± 0.001
3	1.1	C_7_H_6_O_5_	169.0143	169.0142	−0.2156	69.0346; 79.0191; 81.0346; 97.0296; 125.0245	Gallic acid	N/A
4	1.98	C_15_H_14_O_7_	305.0667	305.0667	−0.0220	109.0296; 125.0245; 137.0245; 167.0351; 219.0663	(-)-Gallocatechin	N/A
5	4.04	C_9_H_8_O_4_	179.0350	179.0350	−0.0809	89.0398; 107.0503; 134.0375; 135.0454; 179.0353	Caffeic acid	<LOQ
6	5.58	C_27_H_30_O_15_	593.1512	593.1517	0.8077	183.0452; 255.0299; 283.0248; 430.0905; 447.0933	Kaempferol-3-O-β-glucopyranoside-7-α-rhamnopyranoside	N/A
7	5.77	C_7_H_6_O_3_	137.0244	137.0243	−0.8785	65.0398; 93.0347; 137.0245	Salicylic acid	N/A
8	6.06	C_21_H_20_O_11_	447.0933	447.0937	1.0171	151.0038; 243.0300; 255.0303; 271.0252; 300.0279	Quercitrin (Quercetin-3-O-rhamnoside)	N/A
9	6.82	C_15_H_12_O_5_	271.0612	271.0613	0.1800	65.0033; 83.0140; 107.0139; 119.0503; 151.0038	Naringenin	<LOQ
10	8.08	C_15_H_12_O_4_	255.0663	255.0663	0.0313	65.0034; 83.0141; 107.0139; 151.0038; 171.0451	Pinocembrin	N/A
11	8.32	C_15_H_10_O_4_	253.0506	253.0506	−0.0455	63.0241; 65.0033; 107.0138; 119.0502; 143.0505	Chrysin	N/A

^1^ Quantitative data are expressed as mean ± standard deviation (*n* = 3 independent analysis). Abbreviations: No., compound number; RT, retention time; LOQ, limit of quantification; N/A, not quantified.

**Table 3:** Enzyme inhibitory activity of the aqueous leaf extract of *Lotus rectus* L. (LRAE).

Sample	IC_50_ (µg/mL)			% Inhibition
	Xanthine Oxidase	Tyrosinase	Hyaluronidase	Collagenase
LRAE	93.84 ± 4.44 ^c^	581.01 ± 16.32 ^c^	>600	^##^ 48.93 ± 8.96 ^ns^
ALP	0.36 ± 0.03	-	-	-
EGCG	-	-	-	^# ^61.39 ± 4.04
KA	-	19.66 ± 4.62	-	-
TA	-	-	160.74 ± 16.89	-

Data are expressed as mean ± standard deviation (*n* = 3 independent experiments performed in duplicate). The results are given as percentage of inhibition at a specific concentration (## = 600 μg/mL; # = 200 μg/mL) for the collagenase inhibition assay compared to EGCG (epigallocatechin gallate), while half-maximal inhibitory concentration values (IC_50_) are provided for the xanthine oxidase, tyrosinase and hyaluronidase inhibition assays. Statistical analysis was performed using Student’s *t*-test for independent samples with equal variances or Welch’s *t*-test for samples with unequal variances: ^ns^ *p* ≥ 0.05 and ^c^ *p* < 0.001. Abbreviations: ALP, allopurinol; EGCG, epigallocatechin gallate; KA, kojic acid; TA, tannic acid.

**Table 4:** Fungistatic and fungicidal activity of the aqueous leaf extract of *Lotus rectus* L. (LRAE) against different dermatophytes and yeast strains.

Strains	LRAE		Fluconazole	
	MIC	MLC	MIC	MLC
*Epidermophyton floccosum* FF9	1000	1000	16	16
*Microsporum canis* FF1	800	800	128	128
*Microsporum gypseum* CECT 2908	>1000	-	128	>128
*Trichophyton mentagrophytes* FF7	>1000	-	32	32–64
*Trichophyton mentagrophytes* var. *interdigitale* CECT 2958	>1000	-	128	>128
*Trichophyton rubrum* CECT 2794	>1000	-	16	64
*Trichophyton verrucosum* CECT 2992	>1000	-	>128	-
*Candida krusei* H9	>1000	-	64	64–128
*Candida albicans* ATCC 10231	>1000	-	1	>128
*Candida guilliermondii* MAT23	>1000	-	4	>128
*Candida parapsilosis* ATCC 90018	>1000	-	1	2
*Candida tropicalis* ATCC 13803	>1000	-	4	>128

Data are expressed as μg/mL (*n* = 3 independent experiments performed in duplicate). Ranges of values indicate the variation observed in repeated assays. Abbreviations: MIC, Minimum Inhibitory Concentration; MLC, Minimum Lethal Concentration.

**Table 5:** Calibration data, linearity, LOD and LOQ of quantified compounds in LRAE by UHPLC-HRMS/MS.

Compound	Calibration Curve	Correlation Coefficient (R^2^)	Test Range (µg/mL)	Linear Range (µg/mL)	LOD (µg/mL)	LOQ (µg/mL)
Kaempferol-3,7-O-α-di-rhamnopyranoside	y = 1.269 × 10^5^x + 6.417 × 10^7^	0.995	0.01–10	1–10	0.87	2.64
Hyperoside (Quercetin-3-O-galactoside)	y = 1.778 × 10^5^x + 8.738 × 10^5^	0.997	0.01–10	0.05–1	0.04	0.14
Caffeic acid	y = 5.578 × 10^5^x − 2.677 × 10^6^	0.998	0.01–10	0.01–0.5	0.02	0.05
Naringenin	y = 1.055 × 10^6^x − 7.949 × 10^6^	0.996	0.01–10	0.01–0.2	0.02	0.05

## Data Availability

The original contributions presented in this study are included in the article/Supplementary Materials. Further inquiries can be directed to the corresponding author.

## Supplementary Materials

The following supporting information can be downloaded at https://www.mdpi.com/article/10.3390/plants15091367/s1. Table S1: Expanded Table 2; Document S1: Representative chromatograms of *Lotus rectus* L. (syn. *Dorycnium rectum*) leaf aqueous extract (LRAE) and standards, obtained by UHPLC coupled to a Q-Exactive Orbitrap HRMS. Visualized in Trace Finder v 5.1 and in Xcalibur v 4.3.; Document S2: Representative images of scratch assay with high resolution.

## Abbreviations

The following abbreviations are used in this manuscript:

ABTS	2,2′-azino-bis(3-ethylbenzothiazoline-6-sulfonic acid) diammonium salt
ALP	Allopurinol
ATCC	American Type Culture Collection
CAE	Caffeic acid equivalents
CE	Catechin equivalents
CECT	Spanish Collection of Type Cultures
DPPH	1,1-diphenyl-2-picrylhydrazyl
ECM	Extracellular matrix
EGCG	Epigallocatechin gallate
GAE	Gallic acid equivalents
KA	Kojic acid
LRAE	*Lotus rectus* L. leaf aqueous extract
MIC	Minimum inhibitory concentration
MLC	Minimum lethal concentration
QRT	Quercetin
RE	Rutin equivalents
ROS	Reactive oxygen species
TA	Tannic acid
TRP	Tannin-related phenolics
