# Supplementary material for: Phytochemistry and Wound-Healing, Enzyme-Inhibitory, and Antifungal Activities of the Wild Forage Legume Lotus rectus L
Source: Plants (Basel). 2026 Apr 29;15(9):1367. doi: 10.3390/plants15091367 (PMC13164895; doi:10.3390/plants15091367)
Supplement: Supplementary file 1 [file plants-15-01367-s001.zip › plants-4255734-supplementary.pdf]

Supplementary material. Document S1. Representative chromatograms of *Lotus rectus* L. (syn. *Dorycnium rectum*) leaf aqueous extract (LRAE) and standards, obtained by UHPLC coupled to a Q-Exactive Orbitrap HRMS. Visualized in Trace Finder v 5.1 and in Xcalibur v 4.3.

Authors: González-Vázquez, M., Quílez Guerrero A., Zuzarte, M., Salgueiro L., Alves-Silva, J., De la Puerta R.*
***Corresponding author: puerta@us.es**

*Lotus rectus* L. (syn. _*Dorycnium rectum*_) leaf aqueous extract (LRAE): TIC representative chromatogram. Obtained by UHPLC coupled to a Q-Exactive Orbitrap HRMS. Visualized in Xcalibur v 4.3.

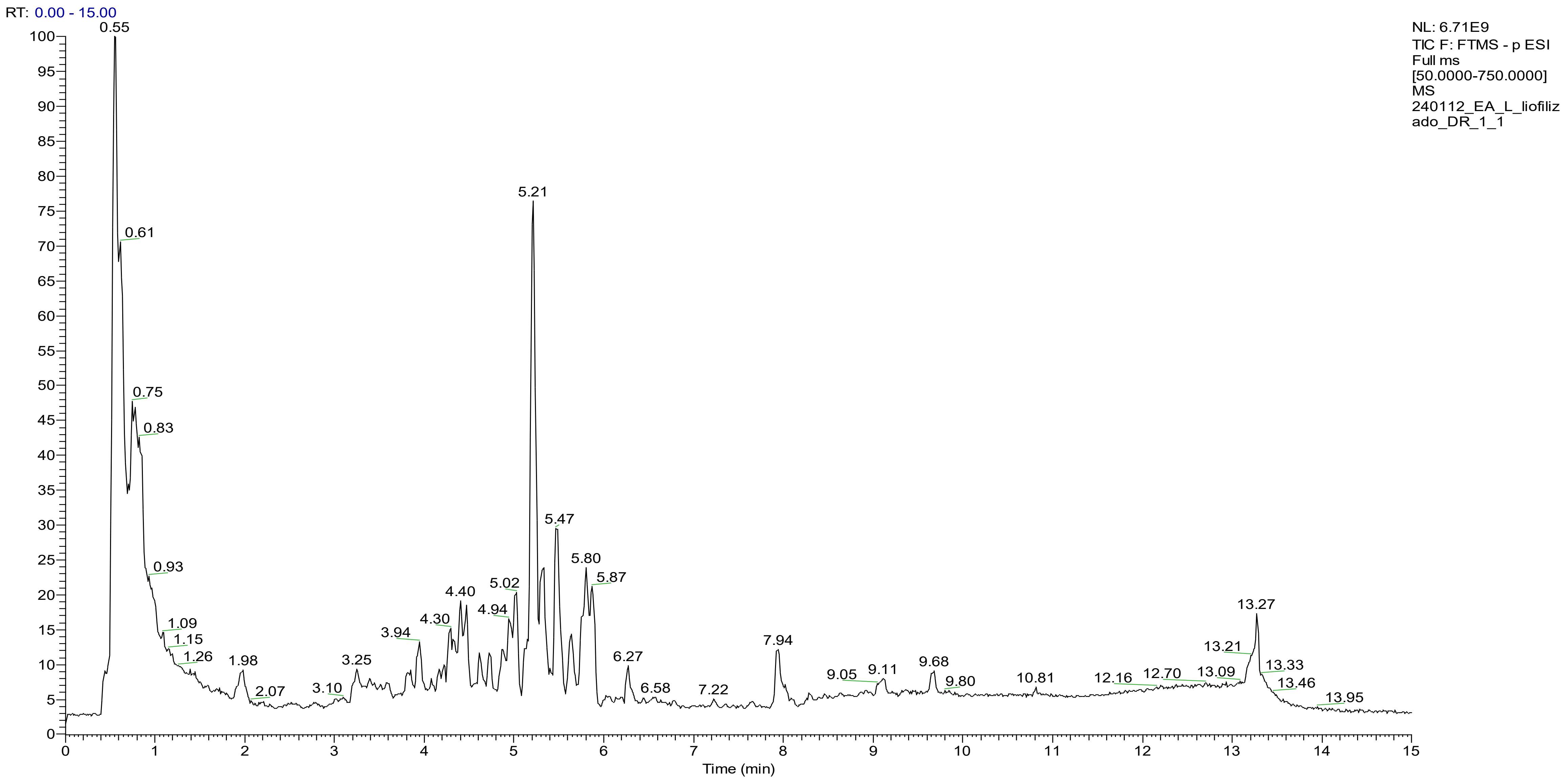

Identification of phenolic compounds by standard comparison (retention times, pseudomolecular ions and fragments ions) against a database of phenolic compounds displayed in Trace Finder v 5.1.

Gallic acid ($C_7H_6O_5$)

Chromatogram

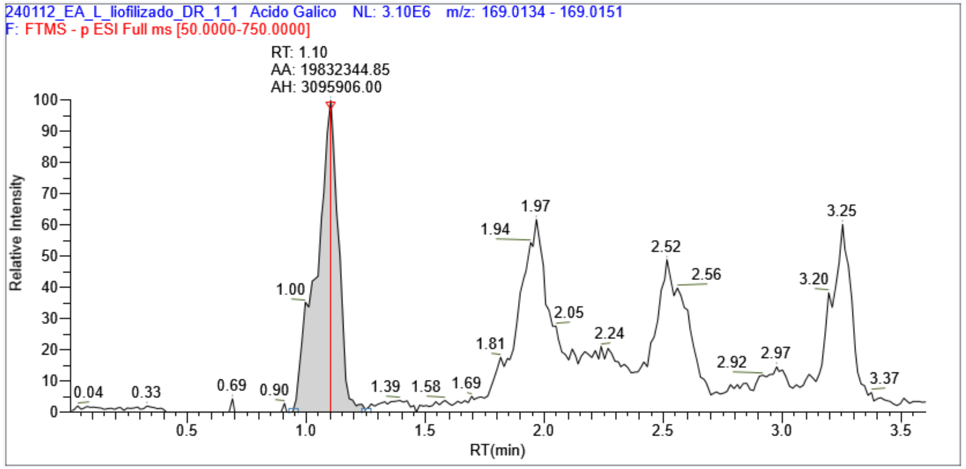

Spectrum

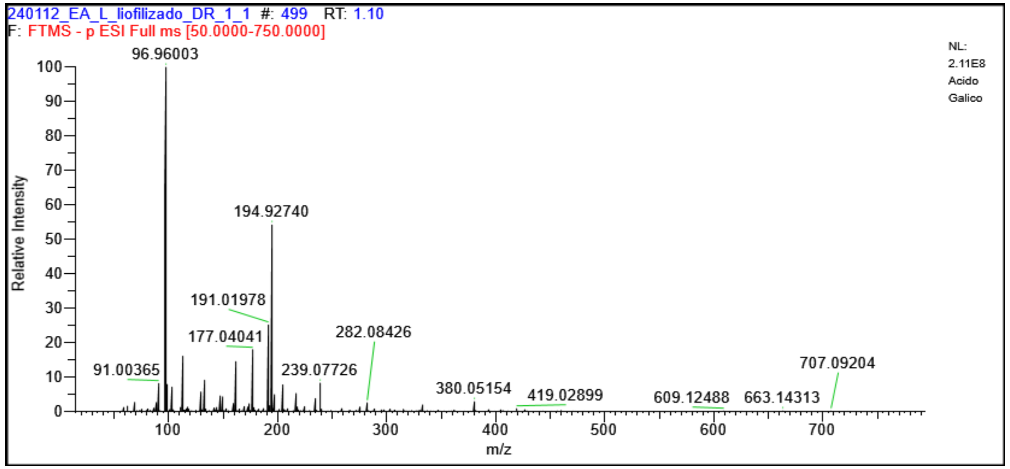

Fragments

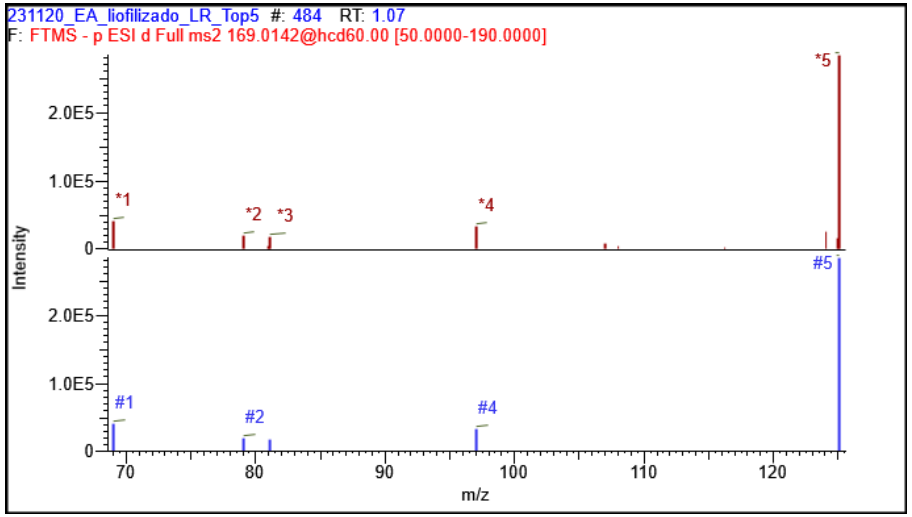

LRAE

Standard

Identification of phenolic compounds by standard comparison (retention times, pseudomolecular ions and fragments ions) against a database of phenolic compounds displayed in Trace Finder v 5.1.

Caffeic acid ($C_9H_8O_4$)

Chromatogram

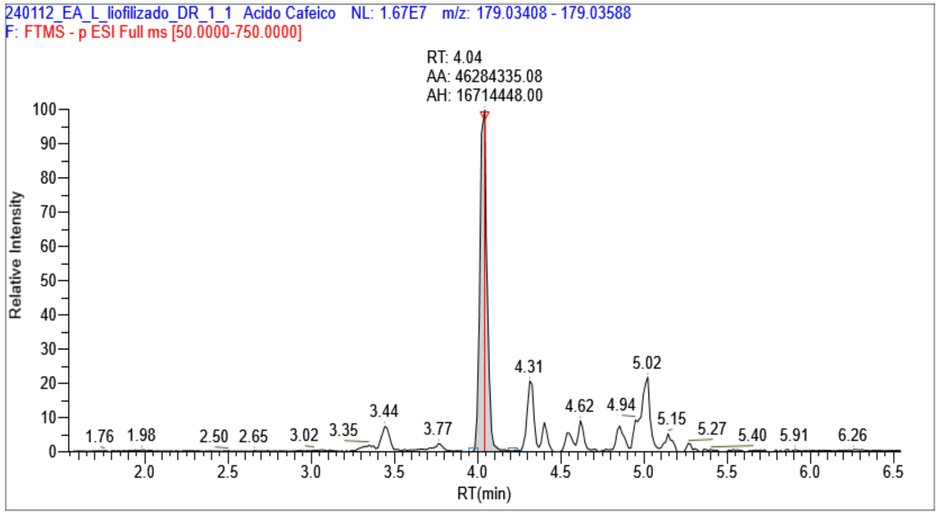

Spectrum

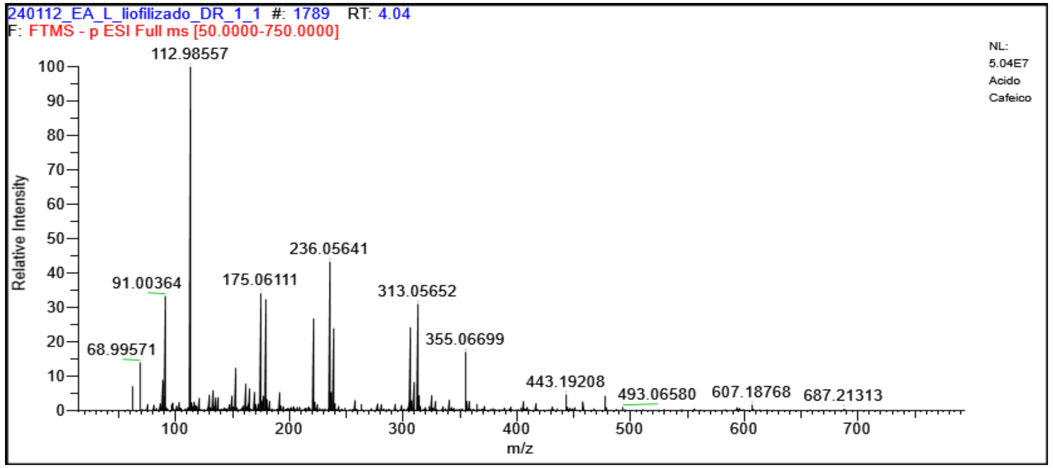

Fragments

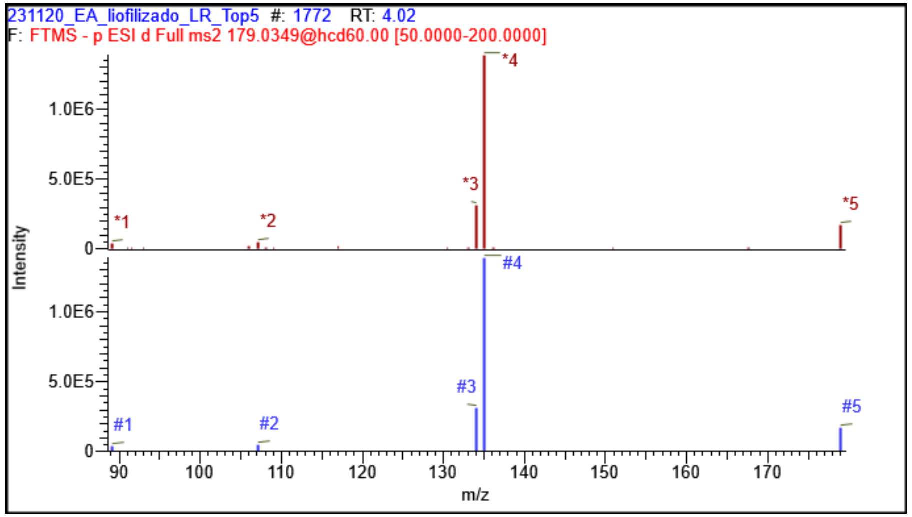

LRAE

Standard

Identification of phenolic compounds by standard comparison (retention times, pseudomolecular ions and fragments ions) against a database of phenolic compounds displayed in Trace Finder v 5.1.

Salicylic acid (C7H6O3)

Chromatogram

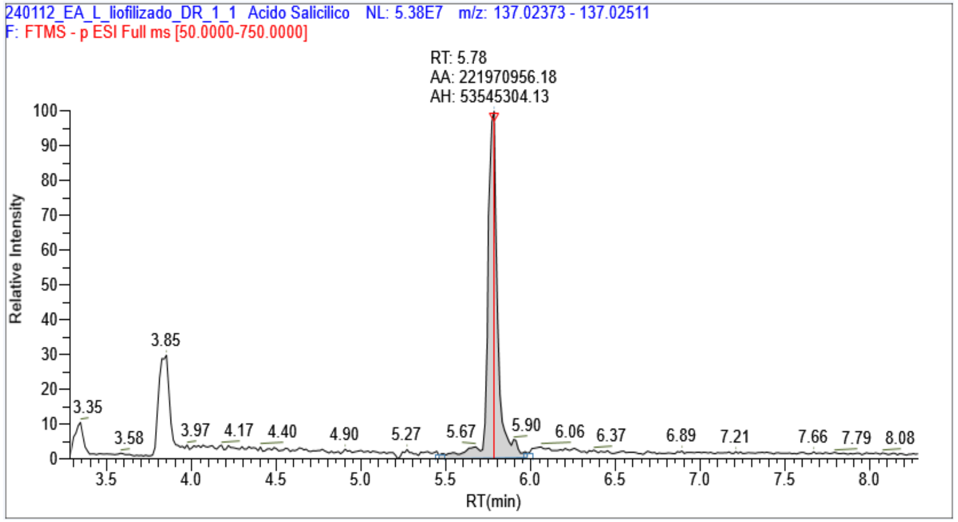

Spectrum

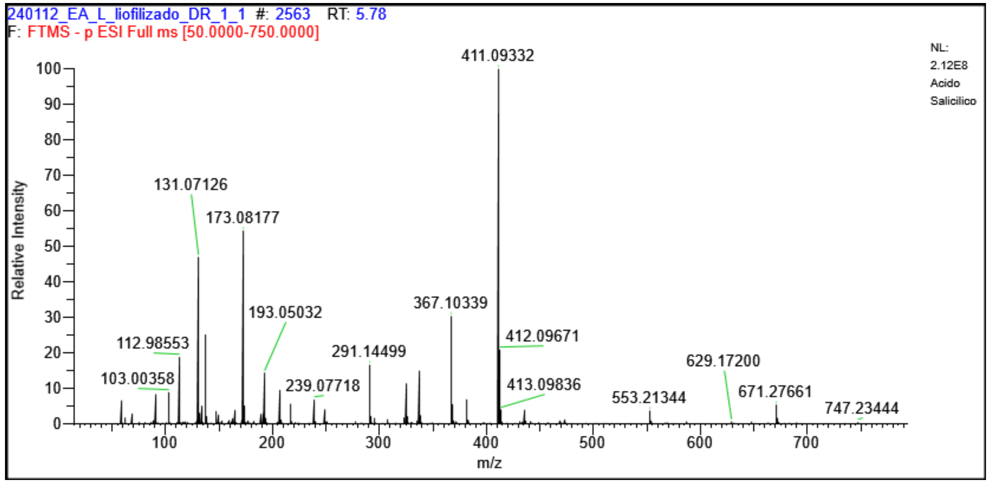

Fragments

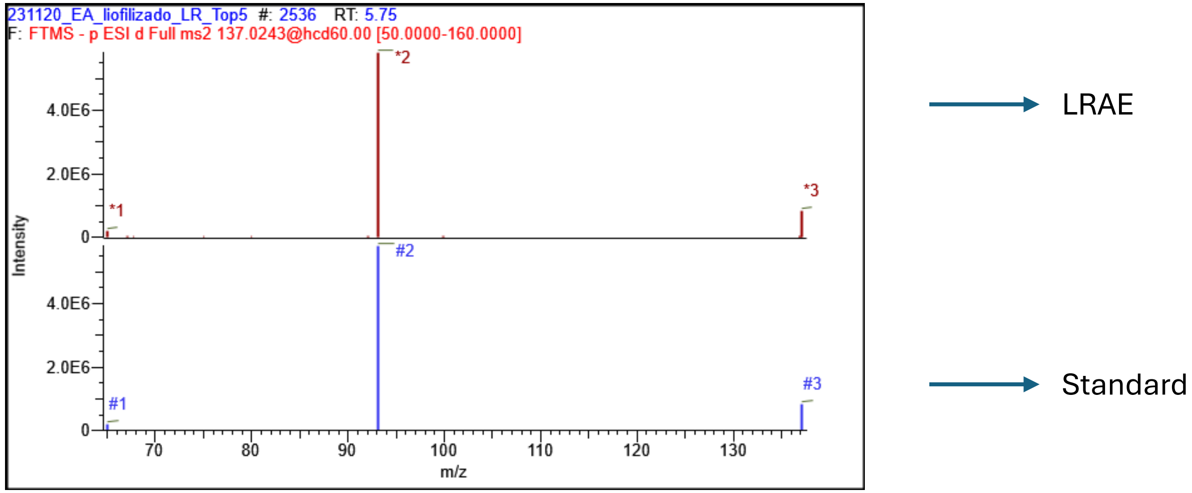

Identification of phenolic compounds by standard comparison (retention times, pseudomolecular ions and fragments ions) against a database of phenolic compounds displayed in Trace Finder v 5.1.

(-)Gallocatechin (C15H14O7)

Chromatogram

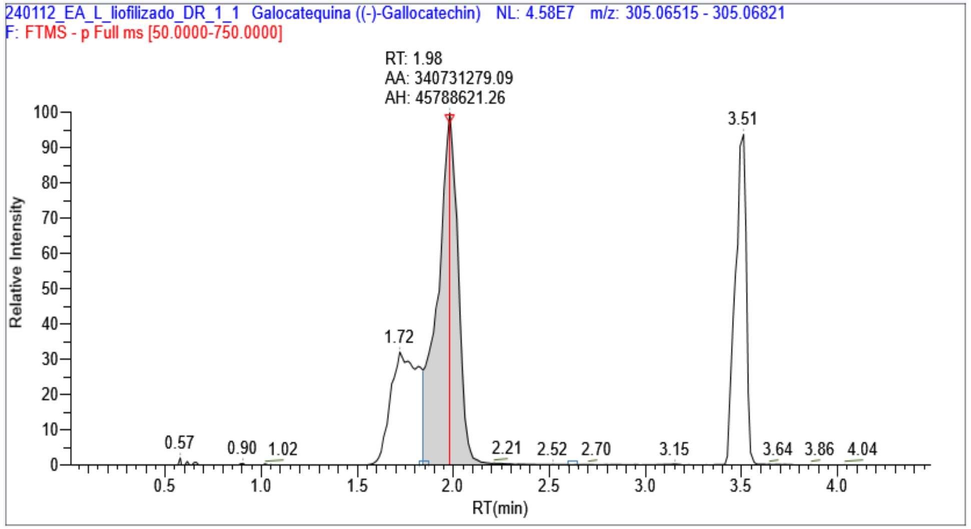

Spectrum

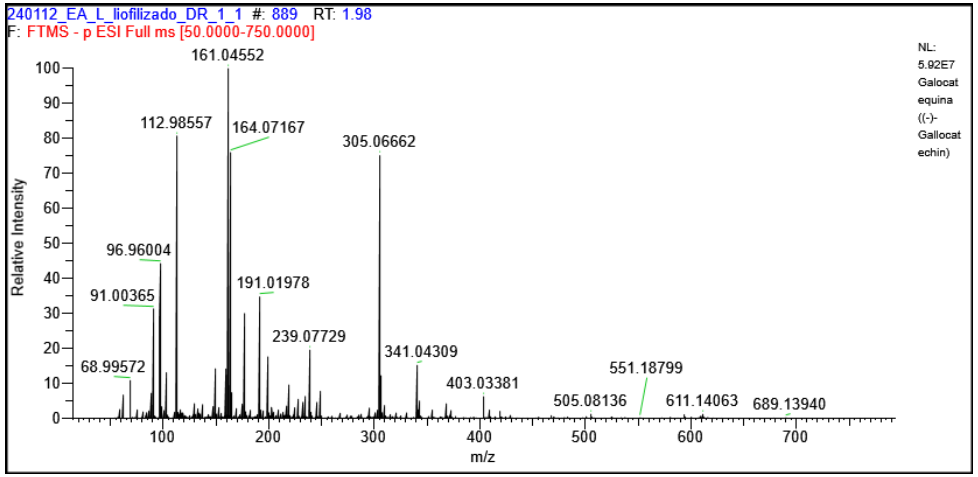

Fragments

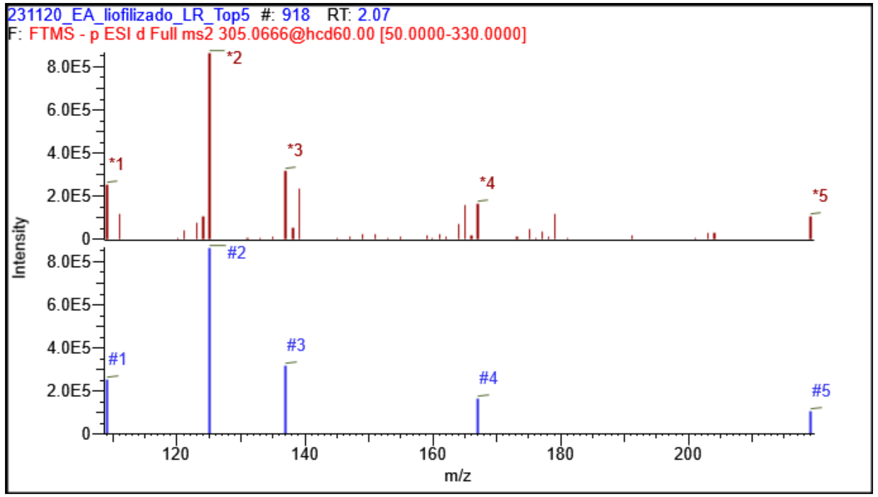

LRAE

Standard

Identification of phenolic compounds by standard comparison (retention times, pseudomolecular ions and fragments ions) against a database of phenolic compounds displayed in Trace Finder v 5.1.

Quercitrin (C21H20O11)

Chromatogram

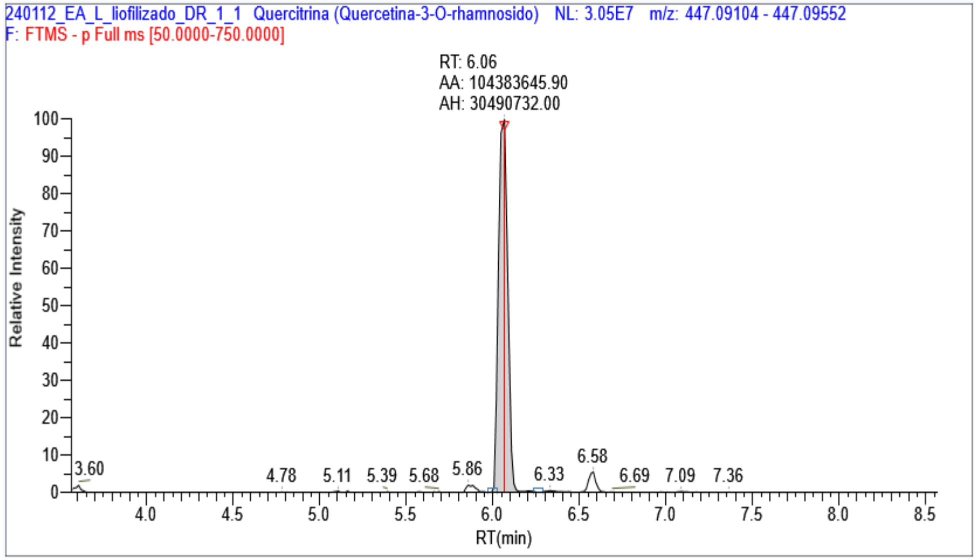

Spectrum

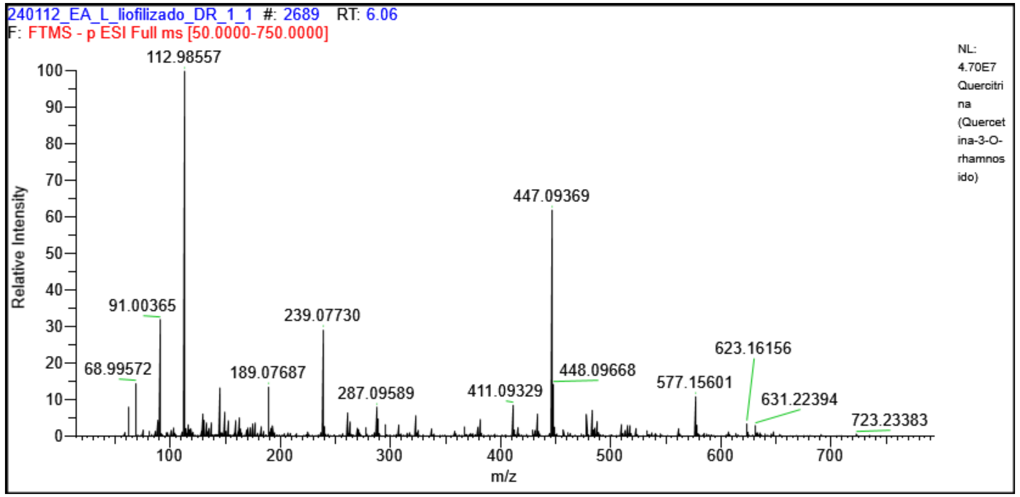

Fragments

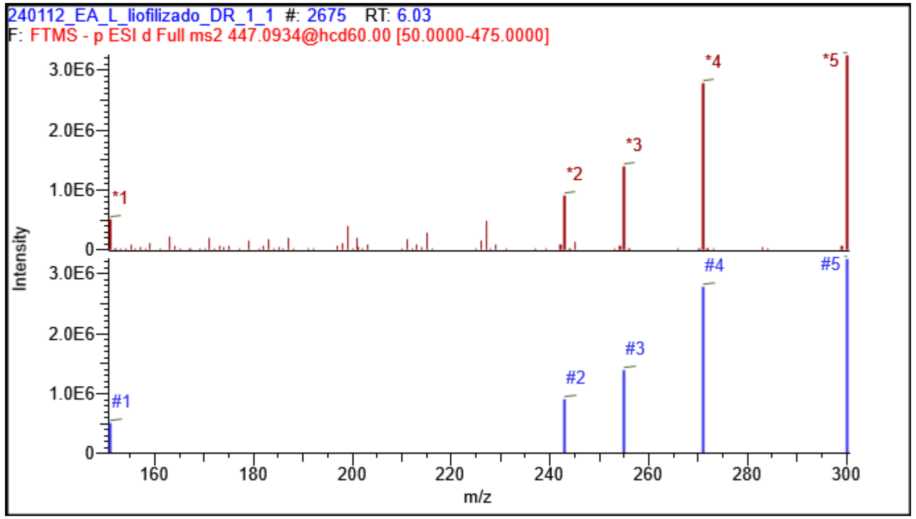

LRAE

Standard

Identification of phenolic compounds by standard comparison (retention times, pseudomolecular ions and fragments ions) against a database of phenolic compounds displayed in Trace Finder v 5.1.

Pinocembrin (C15H12O4)

Chromatogram

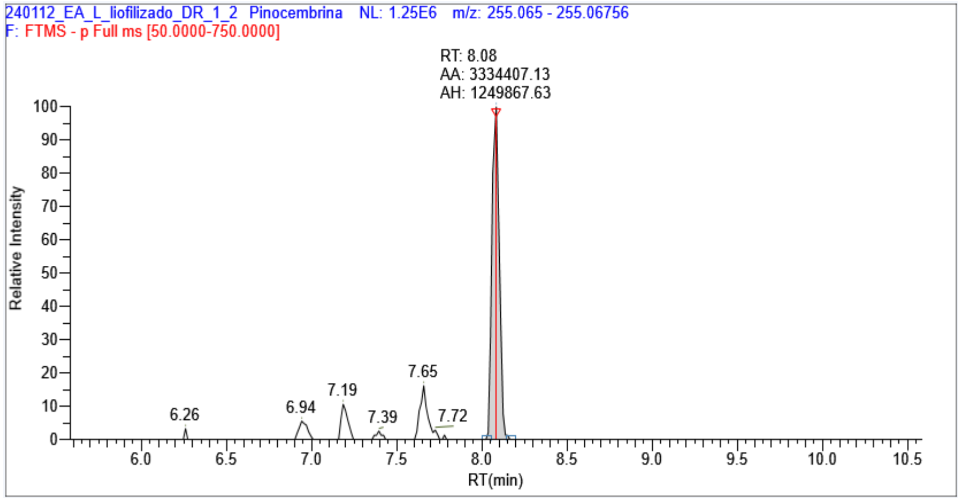

Spectrum

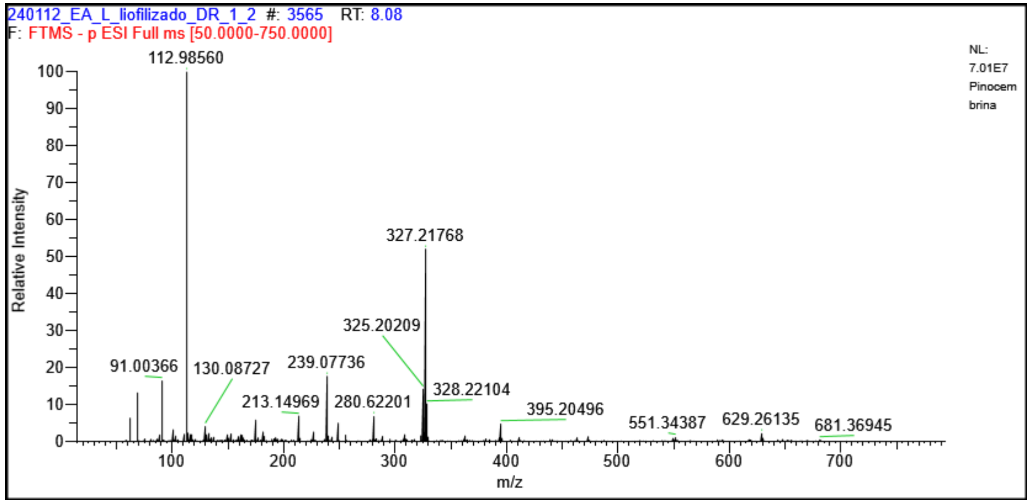

Fragments

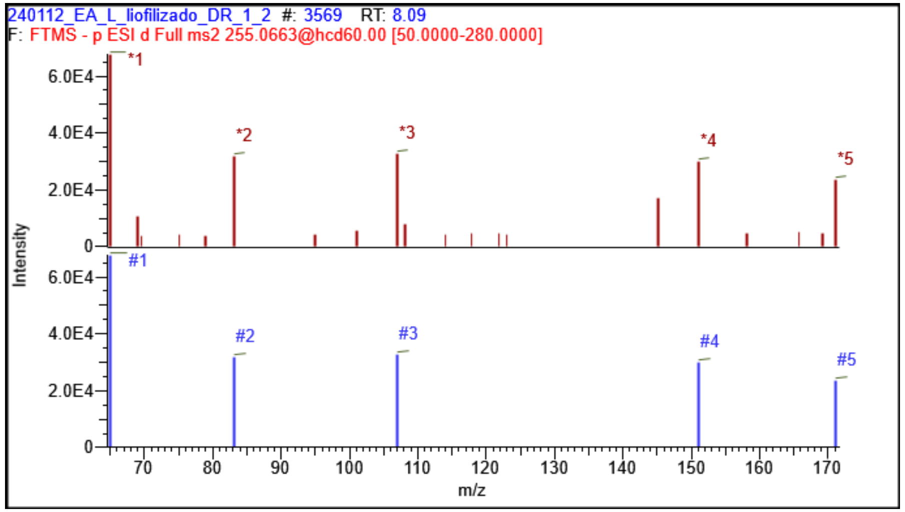

LRAE

Standard

Identification of phenolic compounds by standard comparison (retention times, pseudomolecular ions and fragments ions) against a database of phenolic compounds displayed in Trace Finder v 5.1.

Chrysin ($C_{15}H_{10}O_4$)

Chromatogram

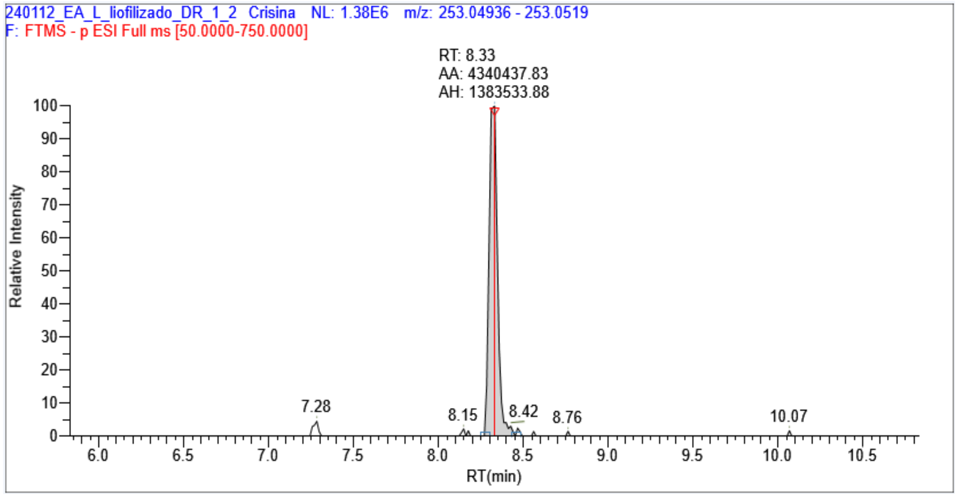

Spectrum

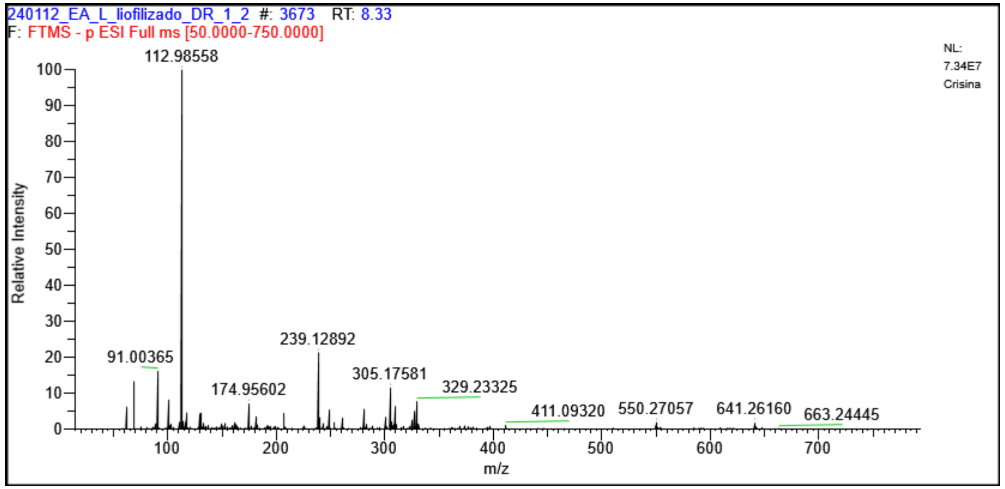

Fragments

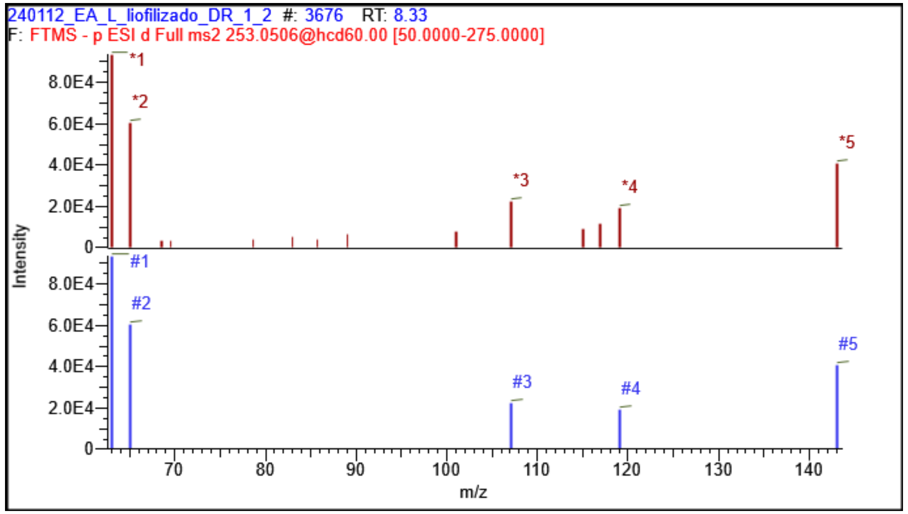

LRAE

Standard

Identification of phenolic compounds by standard comparison (retention times, pseudomolecular ions and fragments ions) against a database of phenolic compounds displayed in Trace Finder v 5.1.

Kaempferol-3,7-O-alpha-di-rhamnopyranoside ($C_{27}H_{30}O_{14}$)

Chromatogram

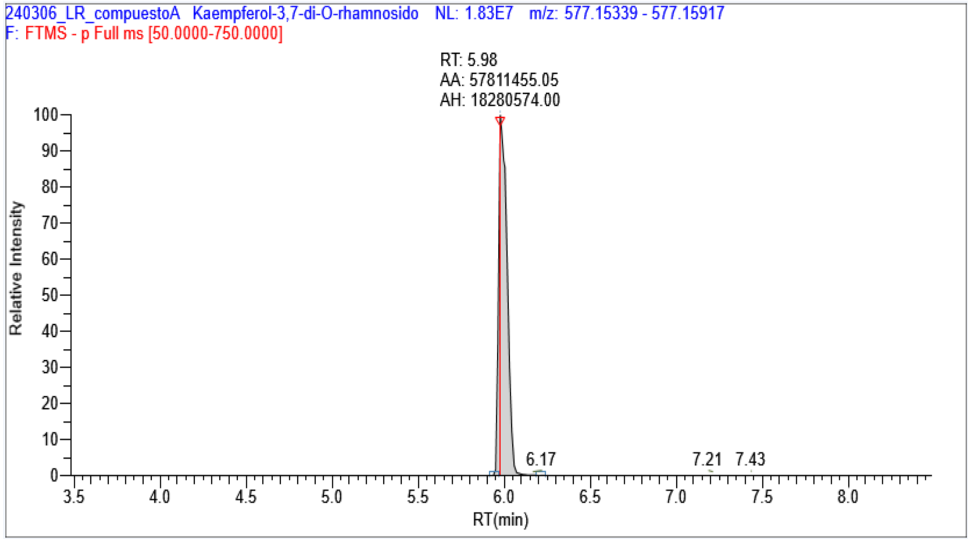

Spectrum

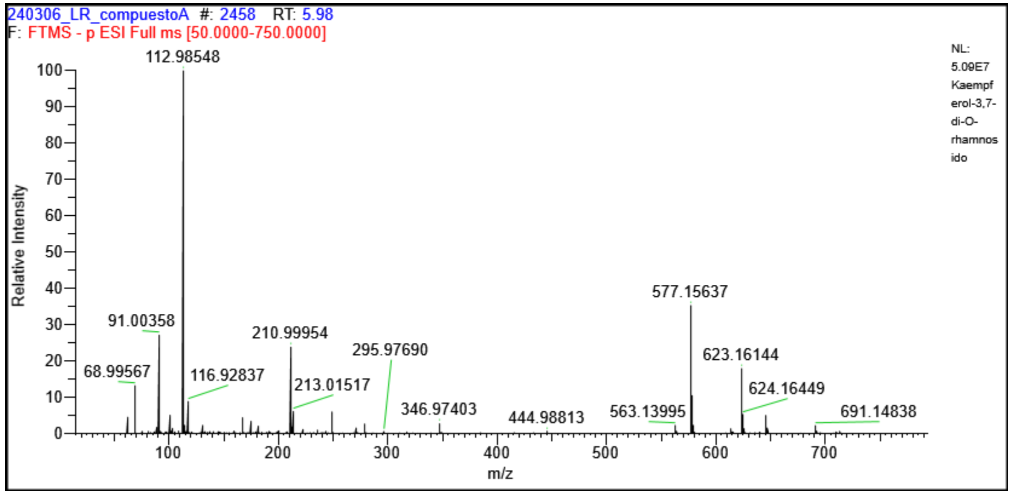

Fragments

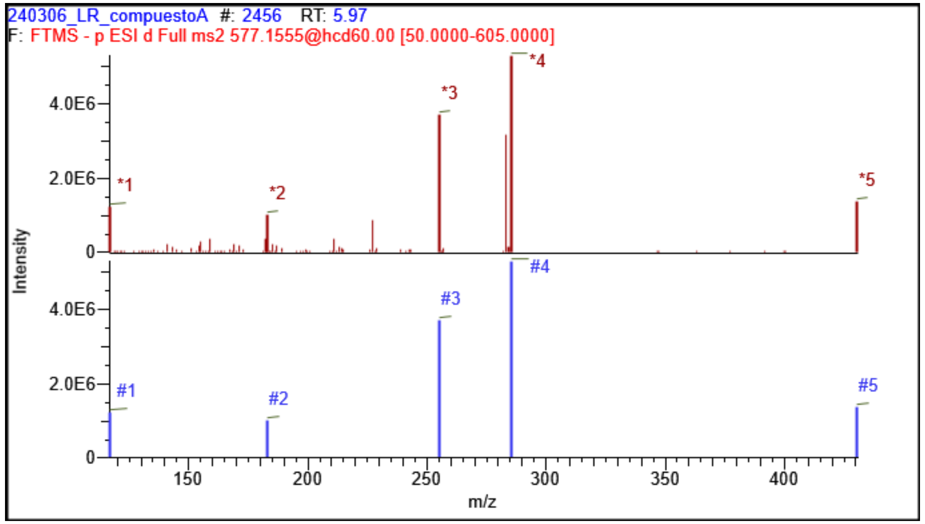

LRAE

Standard

Identification of phenolic compounds by standard comparison (retention times, pseudomolecular ions and fragments ions) against a database of phenolic compounds displayed in Trace Finder v 5.1.

Kaempferol-3-O-beta-glucopyranoside-7-alpha-rhamnopyranoside ($C_{27}H_{30}O_{15}$)

Chromatogram

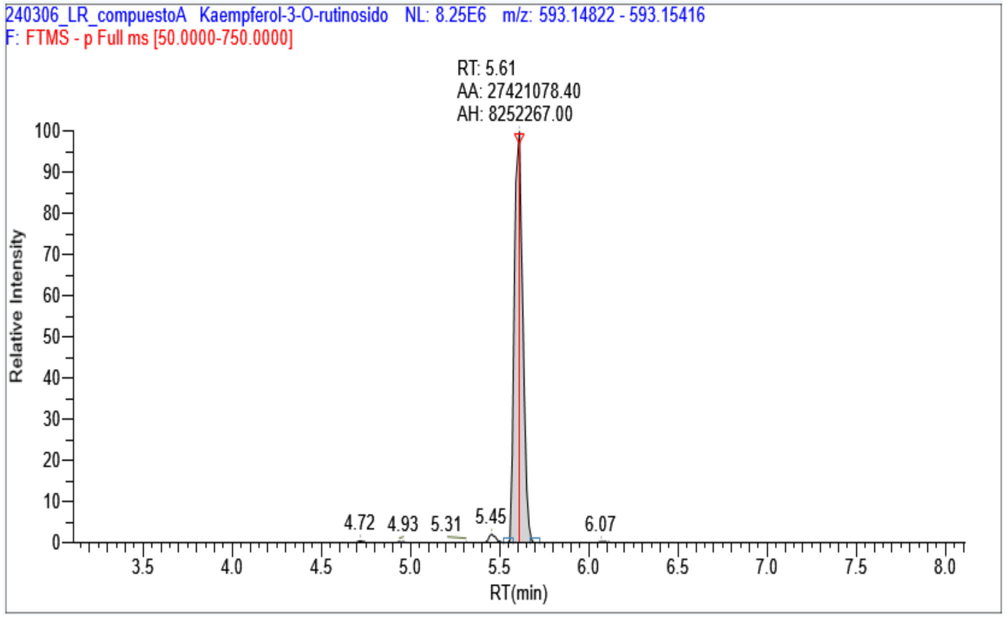

Spectrum

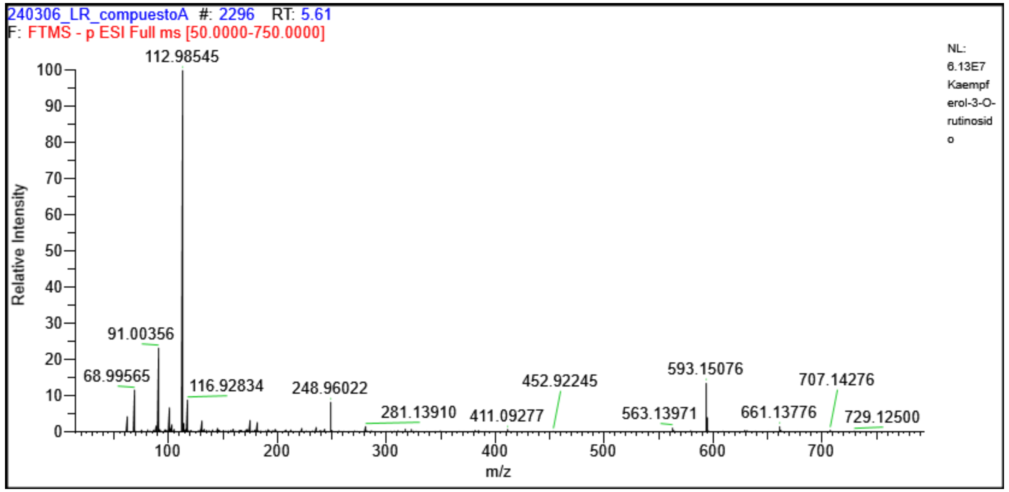

Fragments

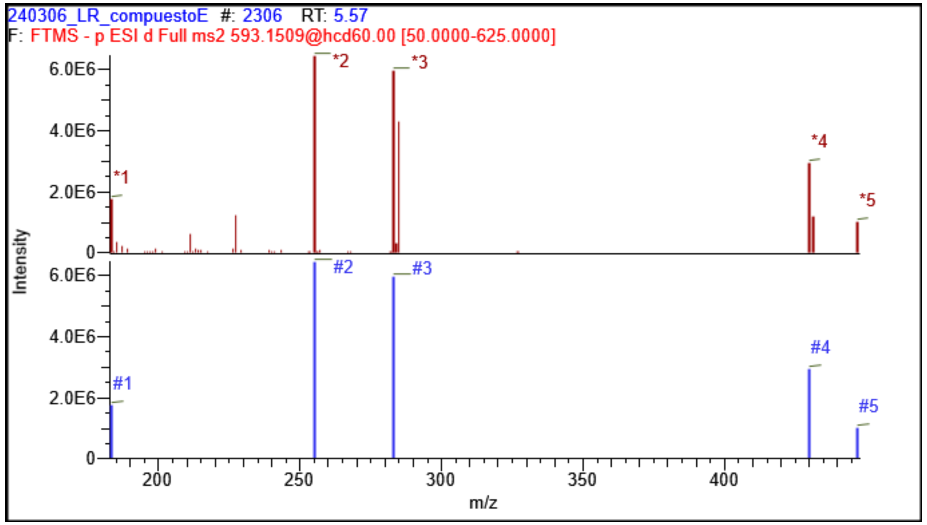

LRAE

Standard

Identification of phenolic compounds by standard comparison (retention times, pseudomolecular ions and fragments ions) against a database of phenolic compounds displayed in Trace Finder v 5.1.

Naringenin ($C_{15}H_{12}O_5$)

Chromatogram (manually integrated in Xcalibur v 4.3)

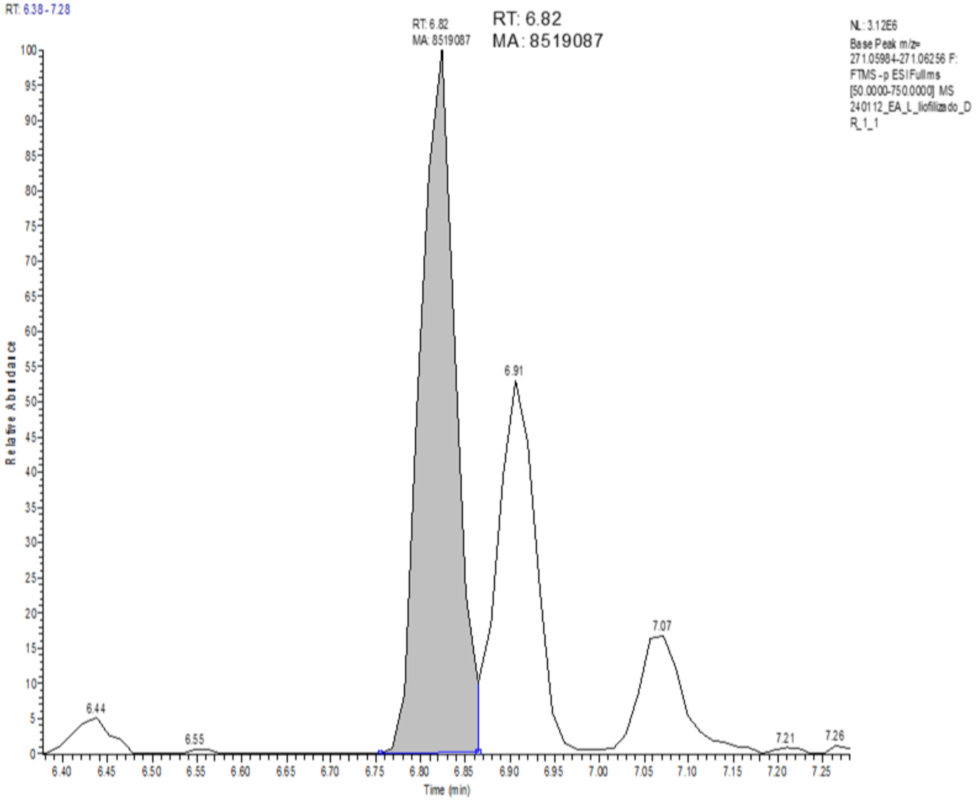

Spectrum

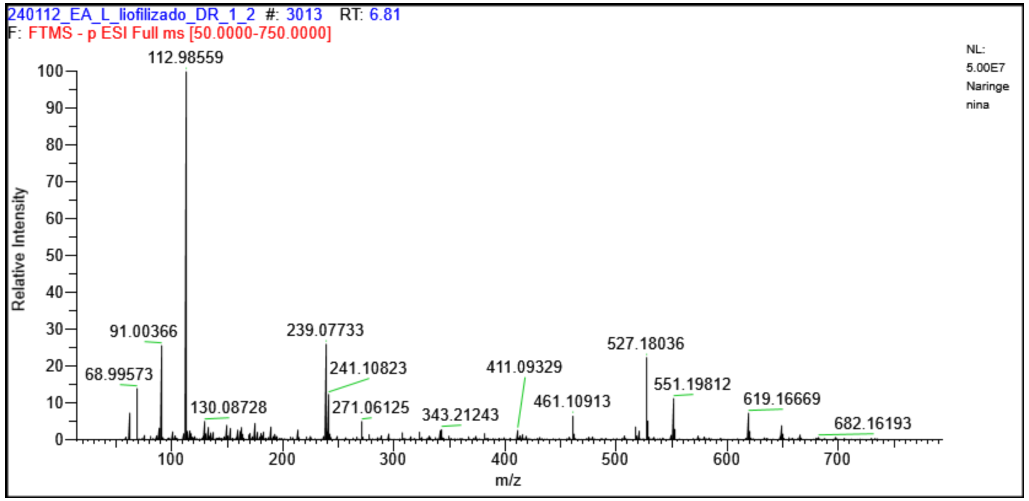

Fragments

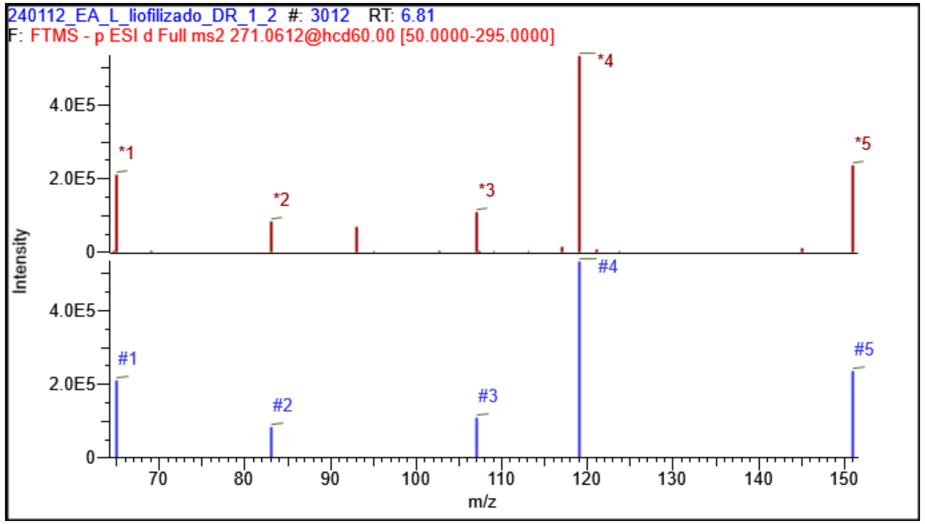

LRAE

Standard

Identification of phenolic compounds by standard comparison (retention times, pseudomolecular ions and fragments ions) against a database of phenolic compounds displayed in Trace Finder v 5.1.

Hyperoside (C21H20O12)

Chromatogram (manually integrated in Xcalibur v 4.3)

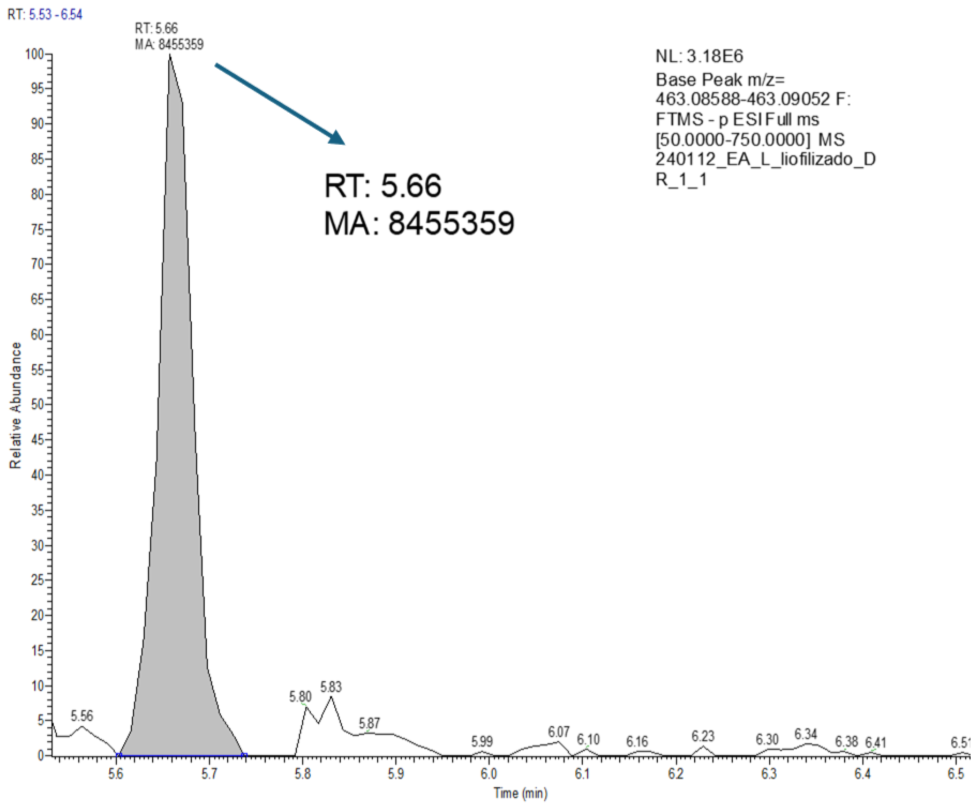

Spectrum

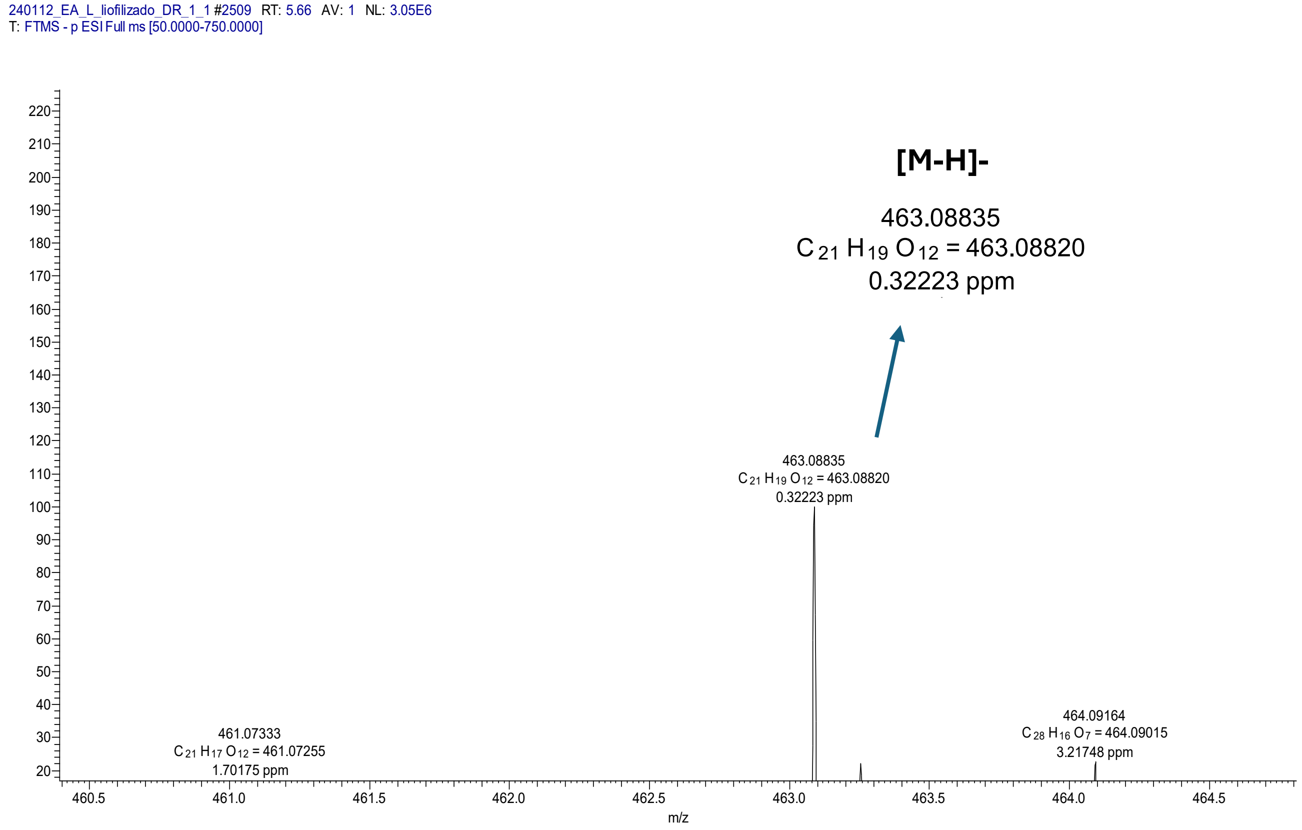

Identification of phenolic compounds by standard comparison (retention times, pseudomolecular ions and fragments ions) against a database of phenolic compounds displayed in Trace Finder v 5.1.

Hyperoside ($C_{21}H_{20}O_{12}$)

Fragments (LRAE)

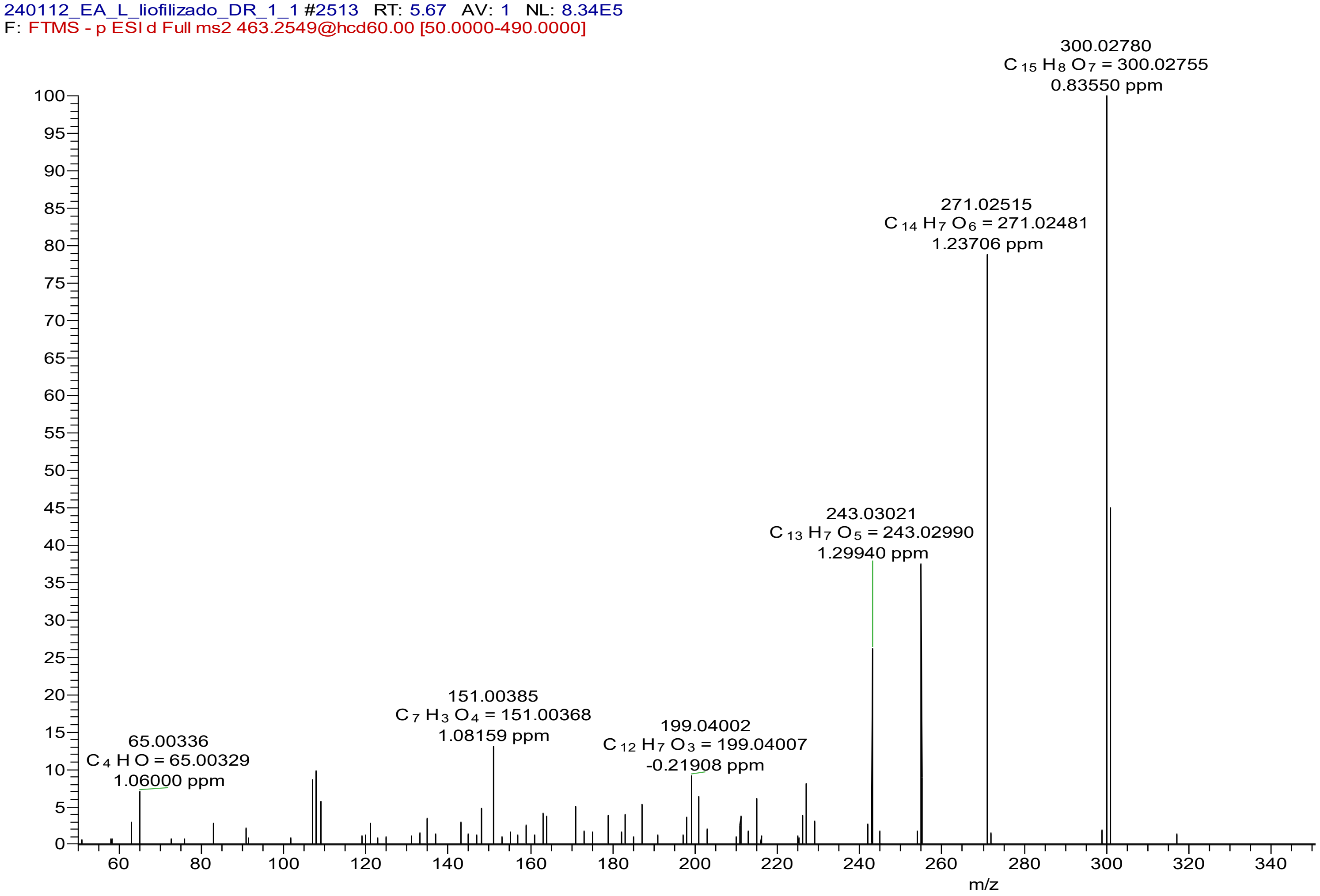

Identification of phenolic compounds by standard comparison (retention times, pseudomolecular ions and fragments ions) against a database of phenolic compounds displayed in Trace Finder v 5.1.

Hyperoside (C21H20O12)

Fragments (LRAE)

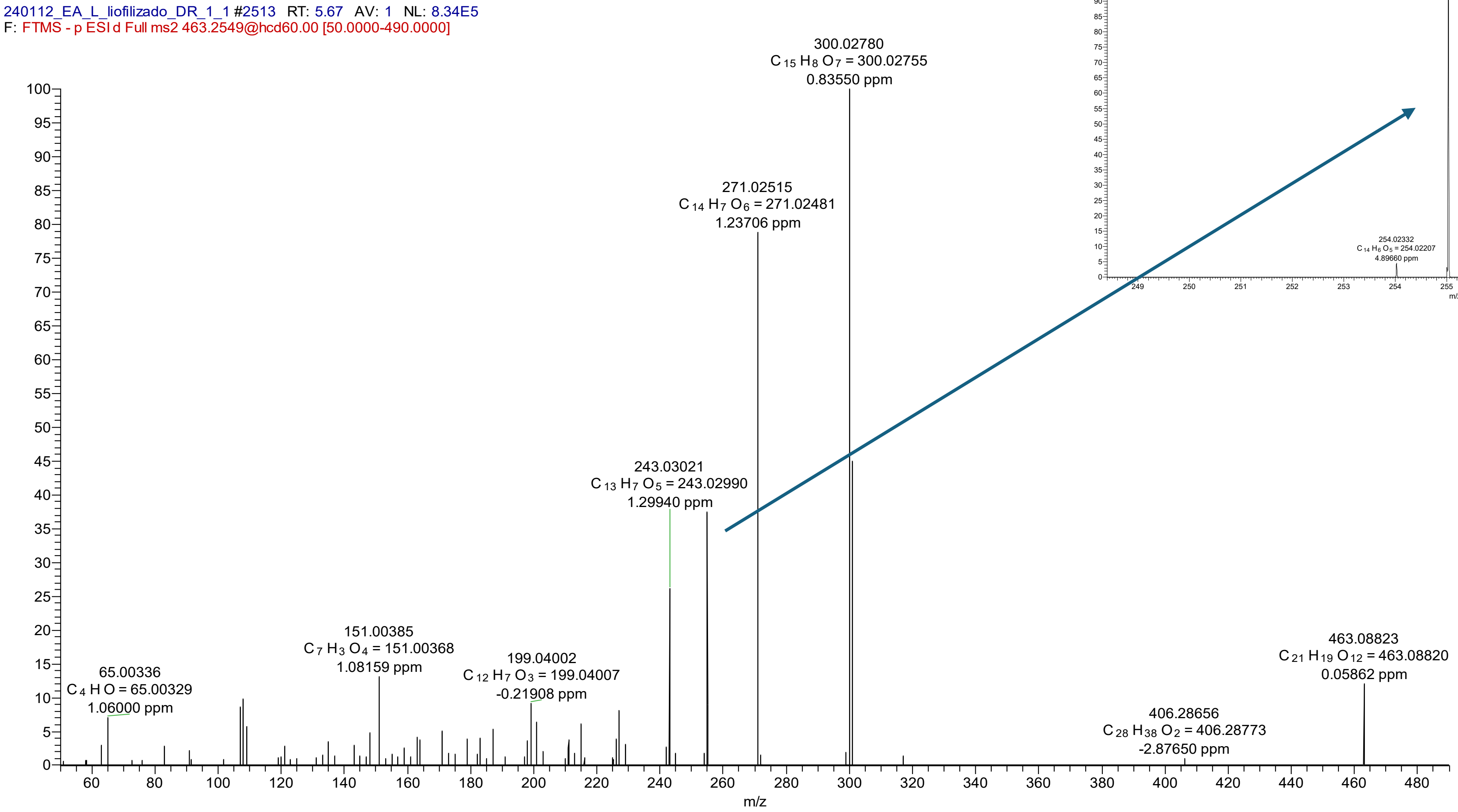

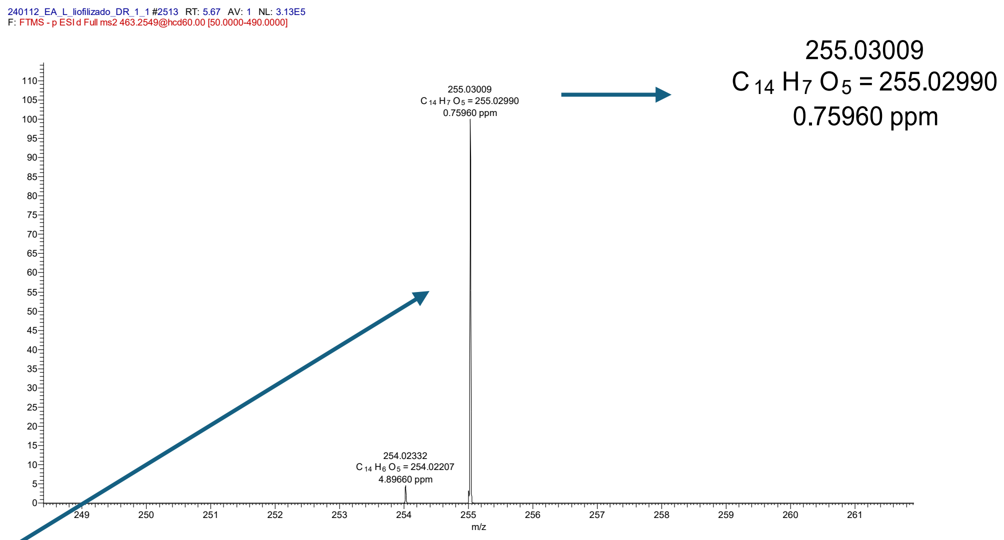

Identification of phenolic compounds by standard comparison (retention times, pseudomolecular ions and fragments ions) against a database of phenolic compounds displayed in Trace Finder v 5.1.

Hyperoside ($C_{21}H_{20}O_{12}$)

Fragments (LRAE)

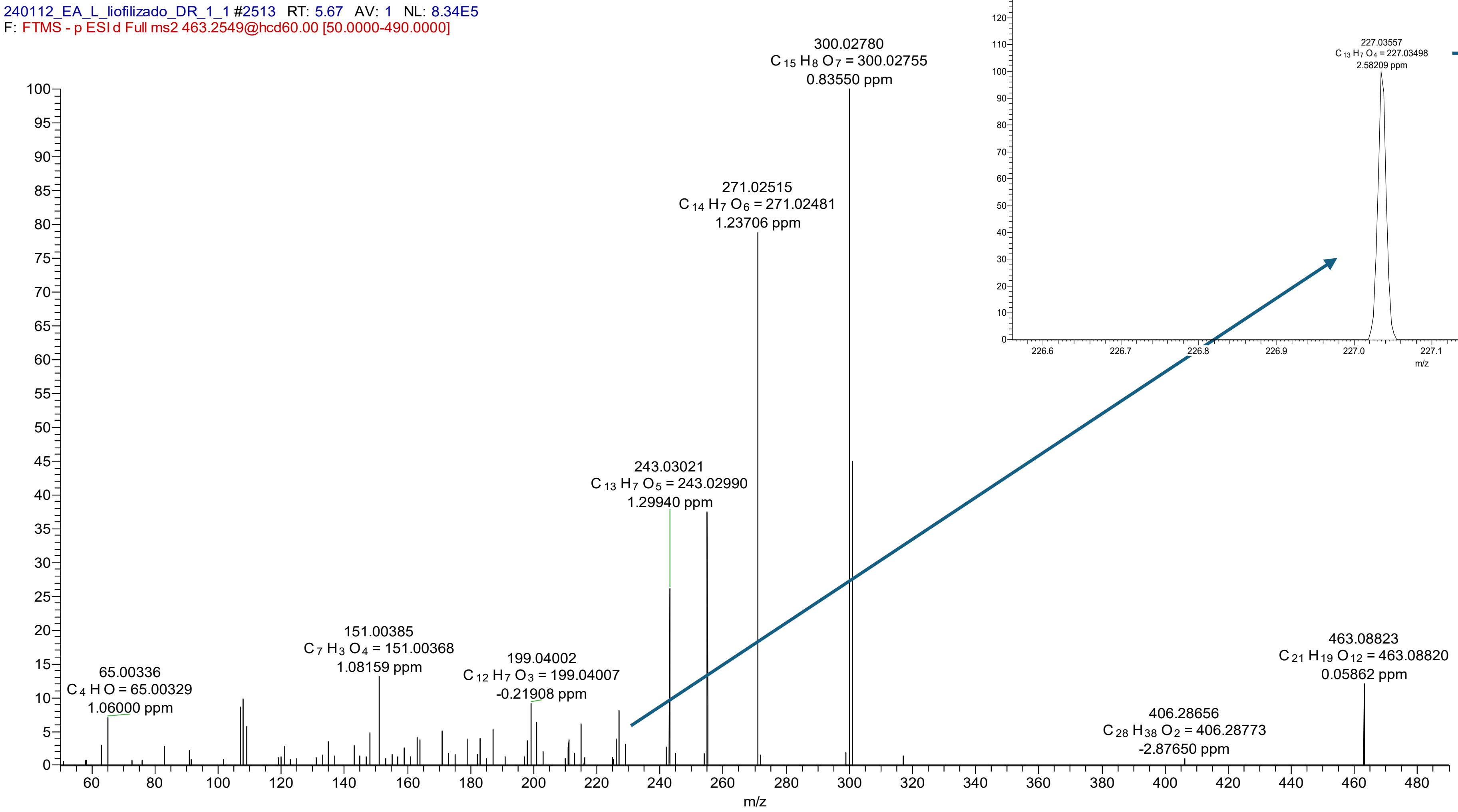

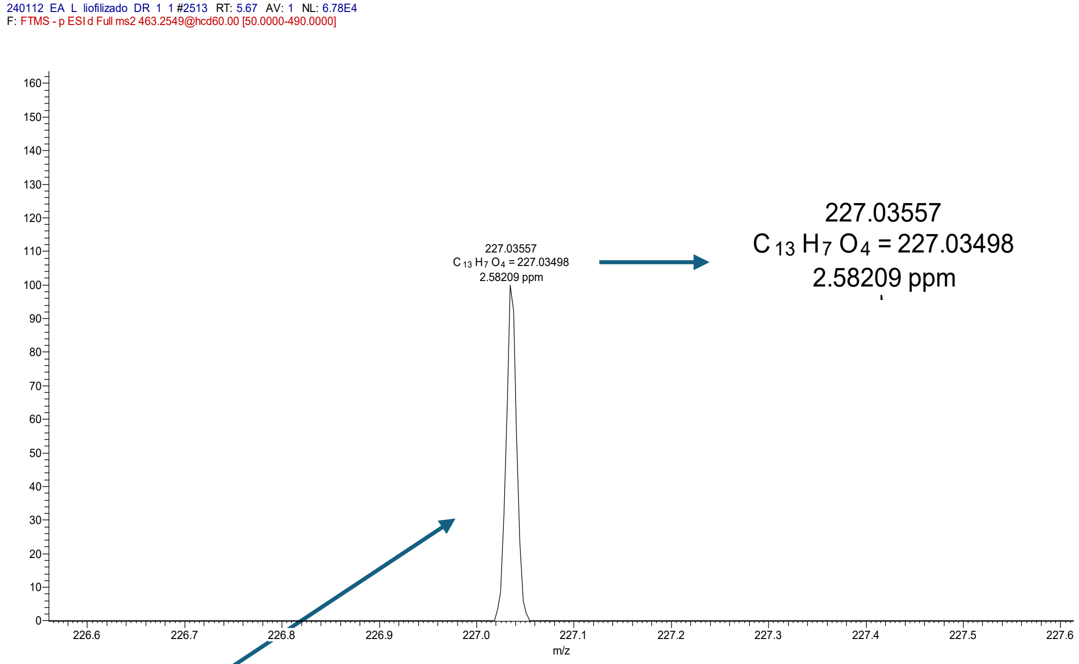

Identification of phenolic compounds by standard comparison (retention times, pseudomolecular ions and fragments ions) against a database of phenolic compounds displayed in Trace Finder v 5.1.

Hyperoside (C21H20O12)

Fragments (Standard) – Xcalibur v 4.3

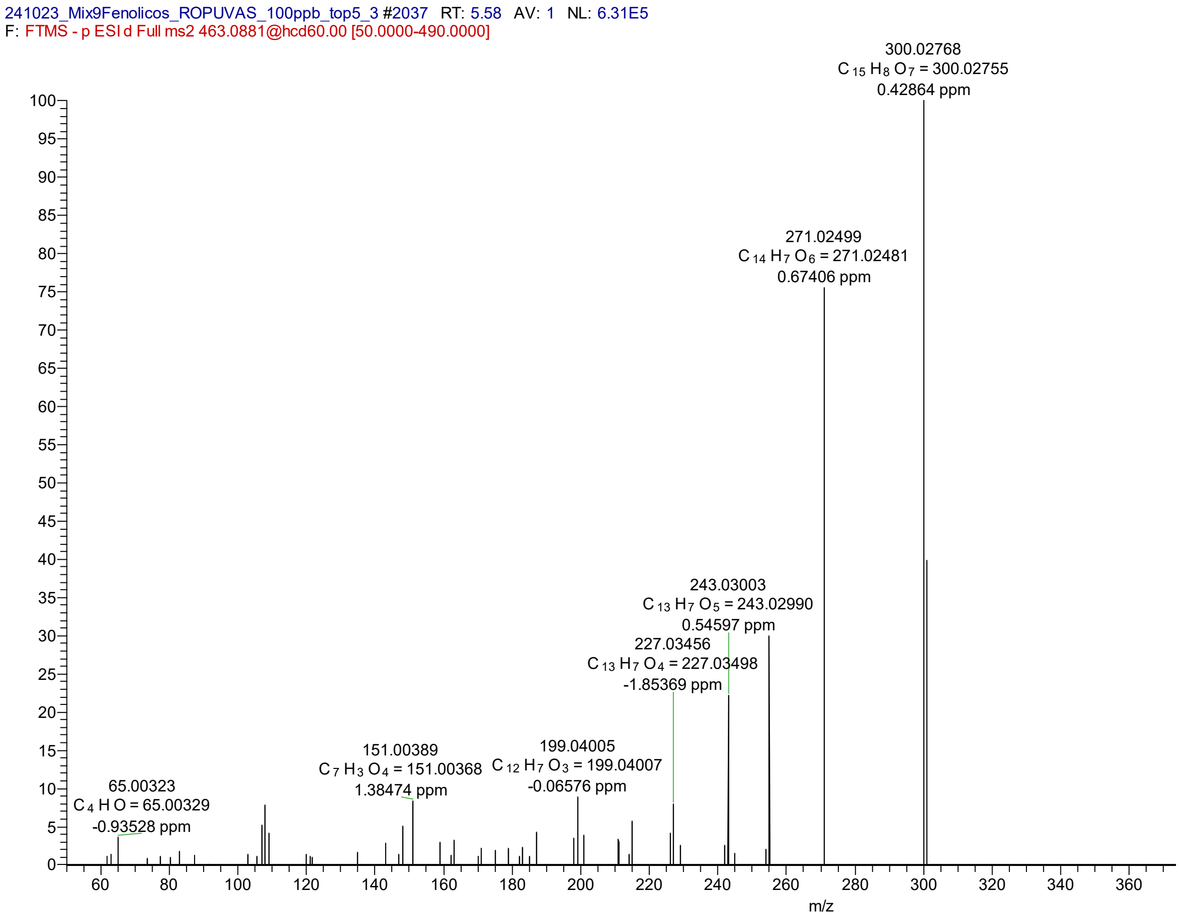

Fragments (Standard) – Trace Finder v 5.1

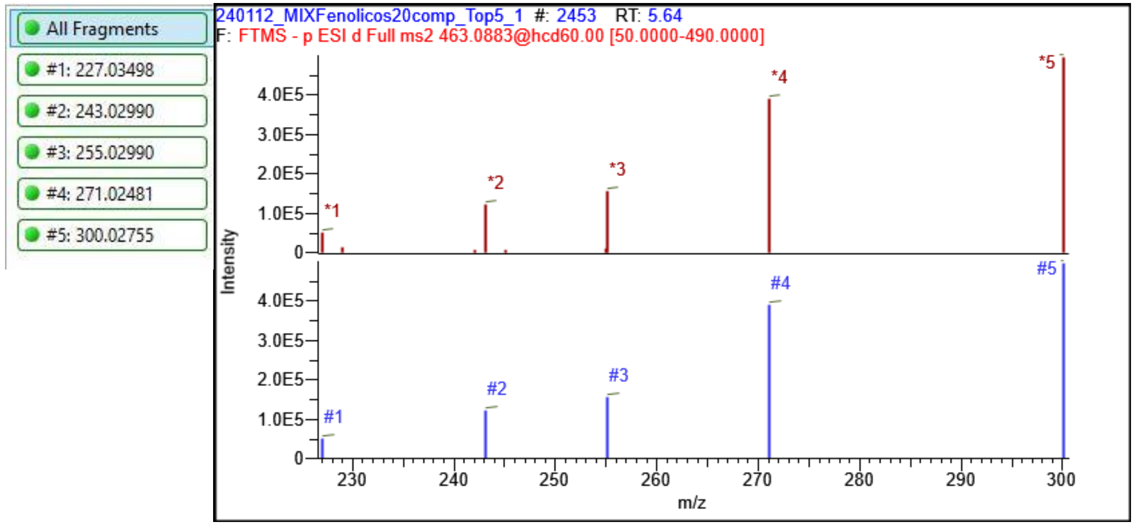

Supplementary material. Document S2. Representative images of scratch assay with high resolution.
Authors: González-Vázquez, M., Quílez Guerrero A., Zuzarte, M., Salgueiro L., Alves-Silva, J., De la Puerta R.*
*Corresponding author: puerta@us.es

- Control 0h

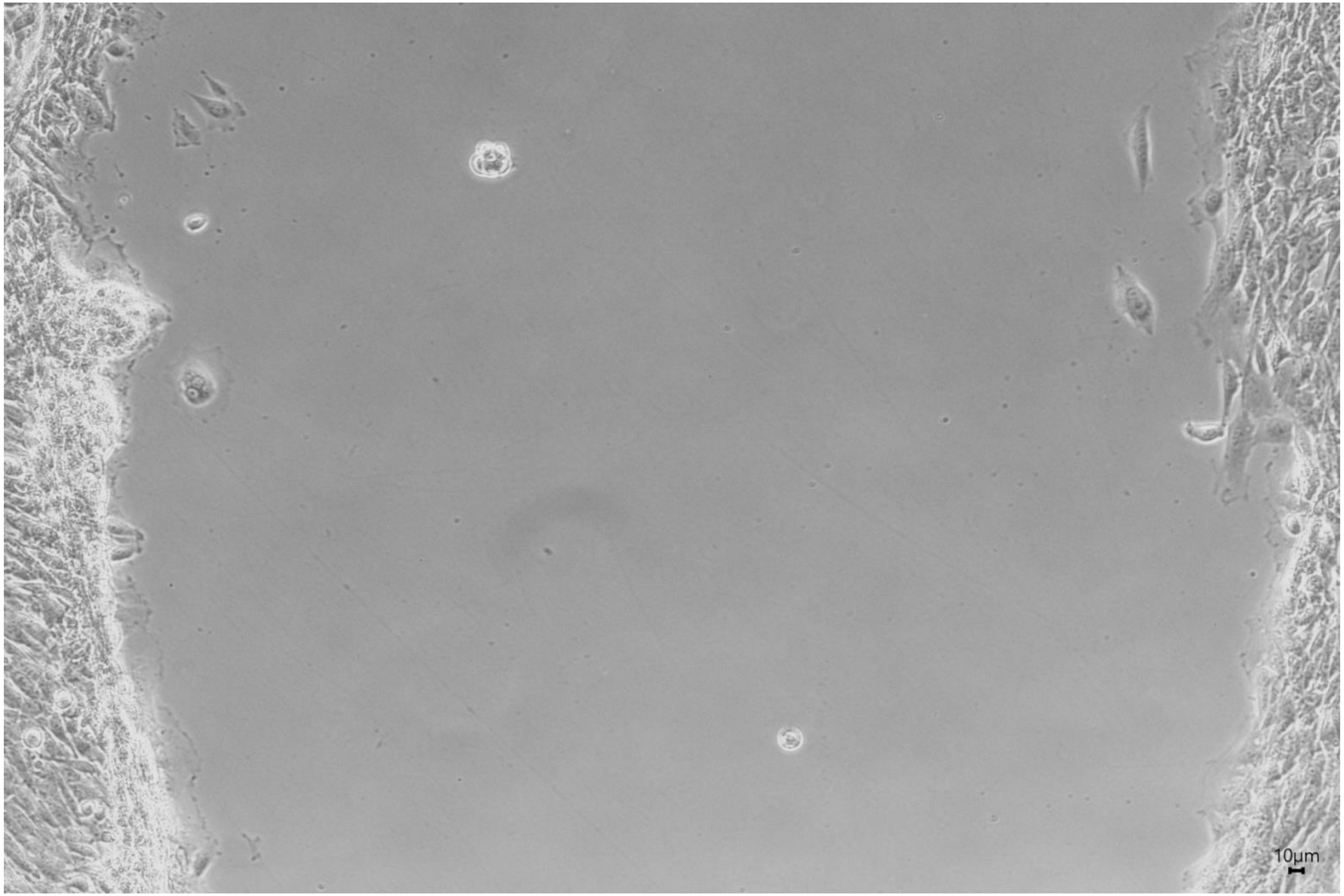

- Control 18h

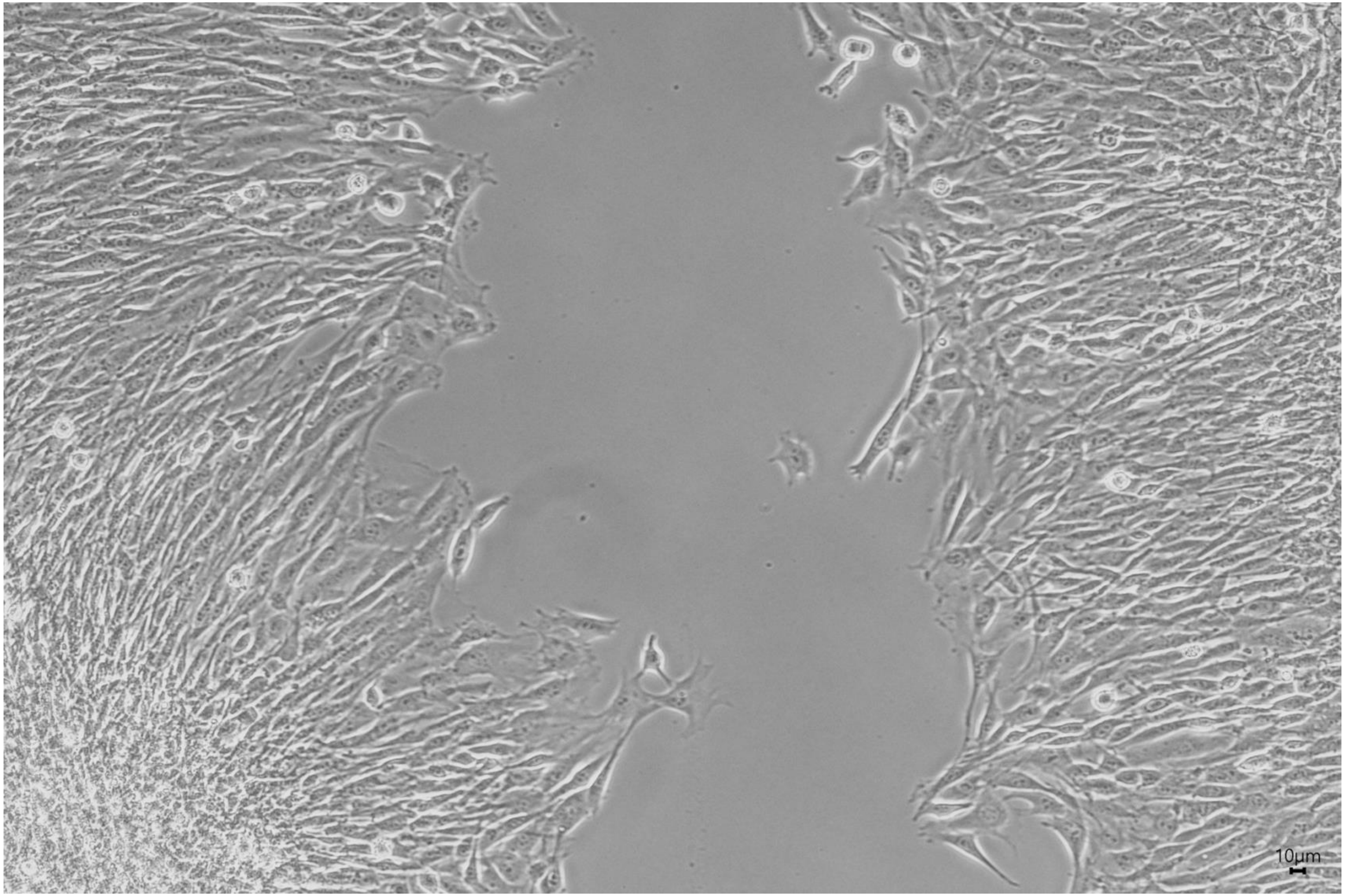

- LRAE 50 μg/mL 0h

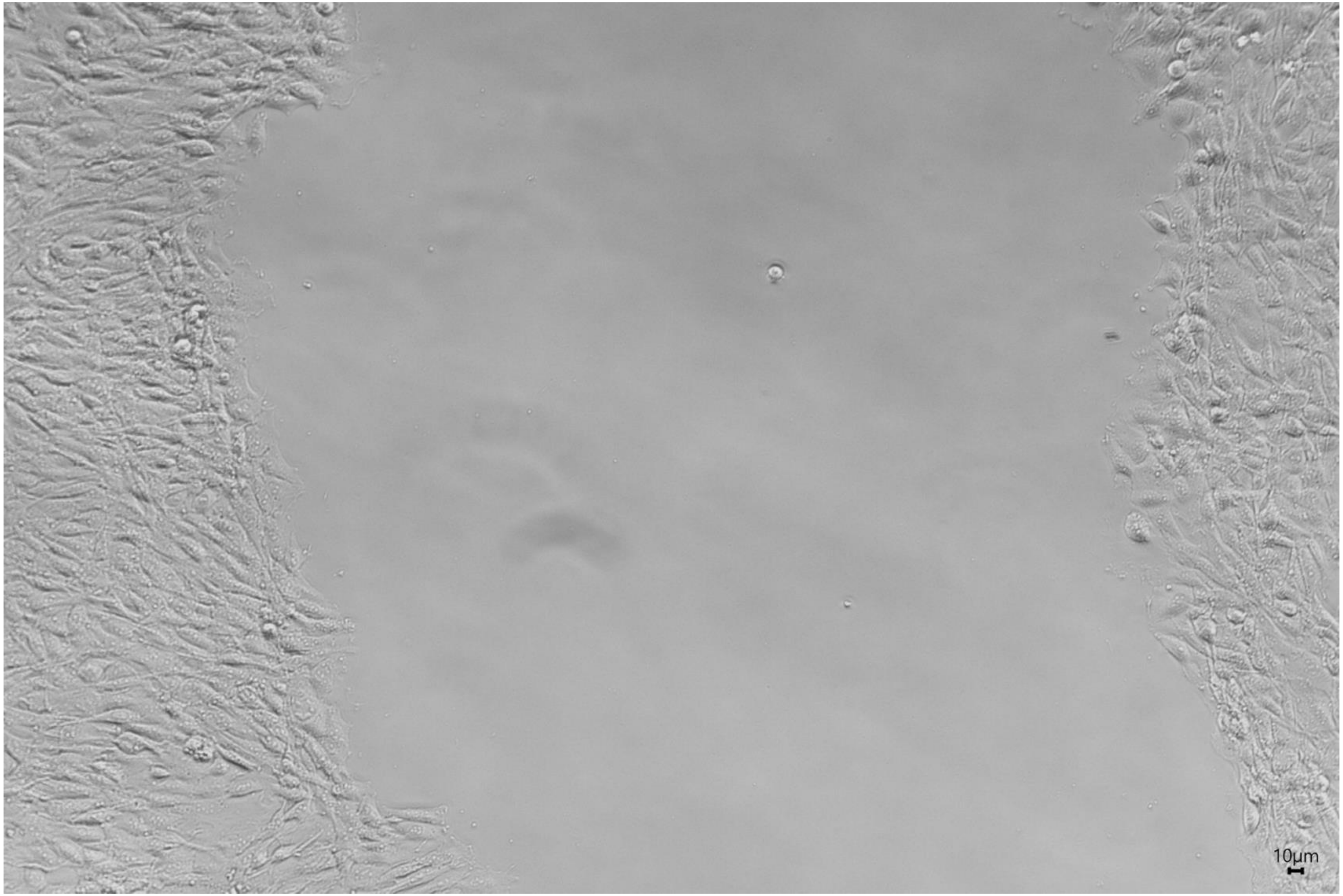

- LRAE 50 µg/mL 18h

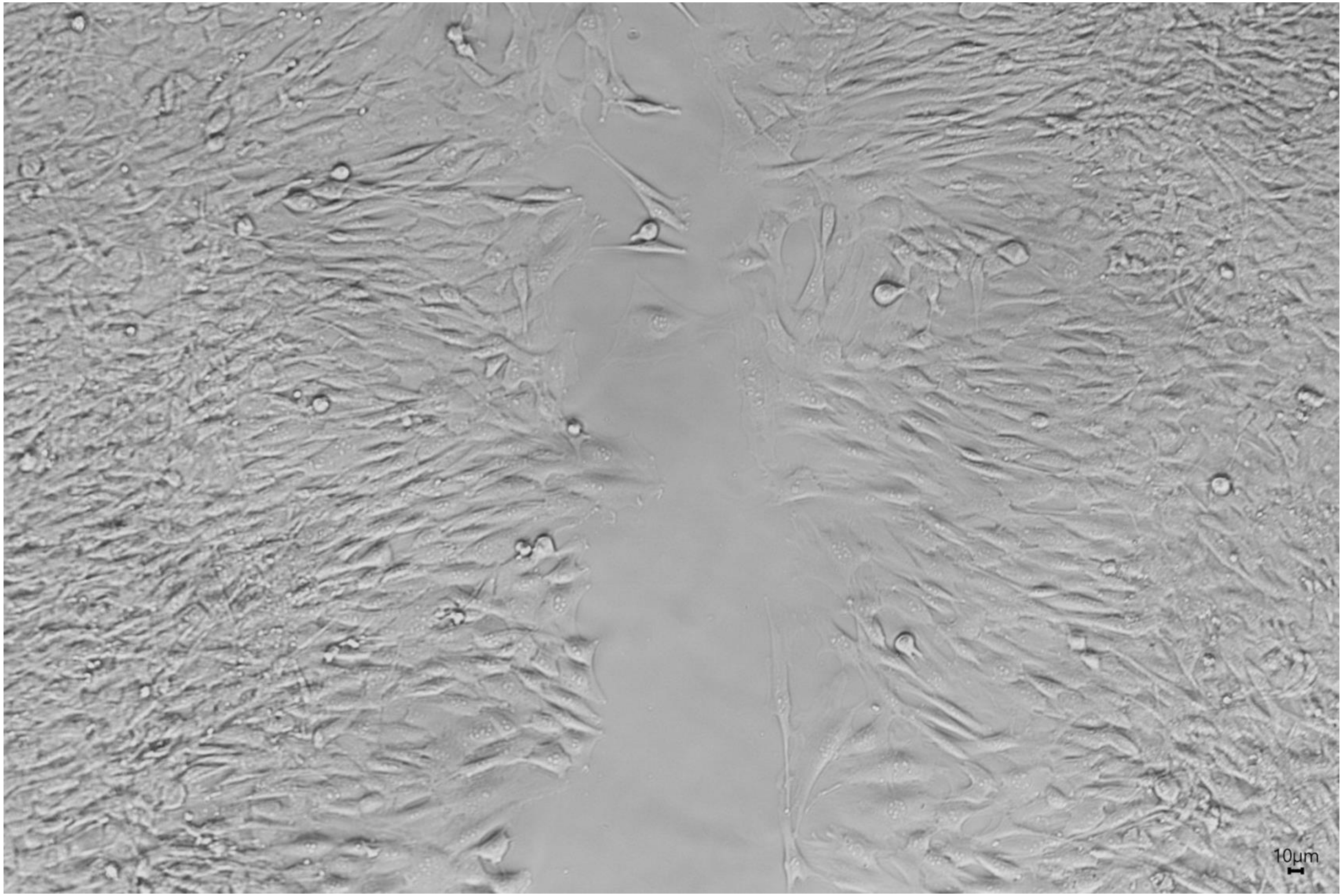

- LRAE 100 μg/mL 0h

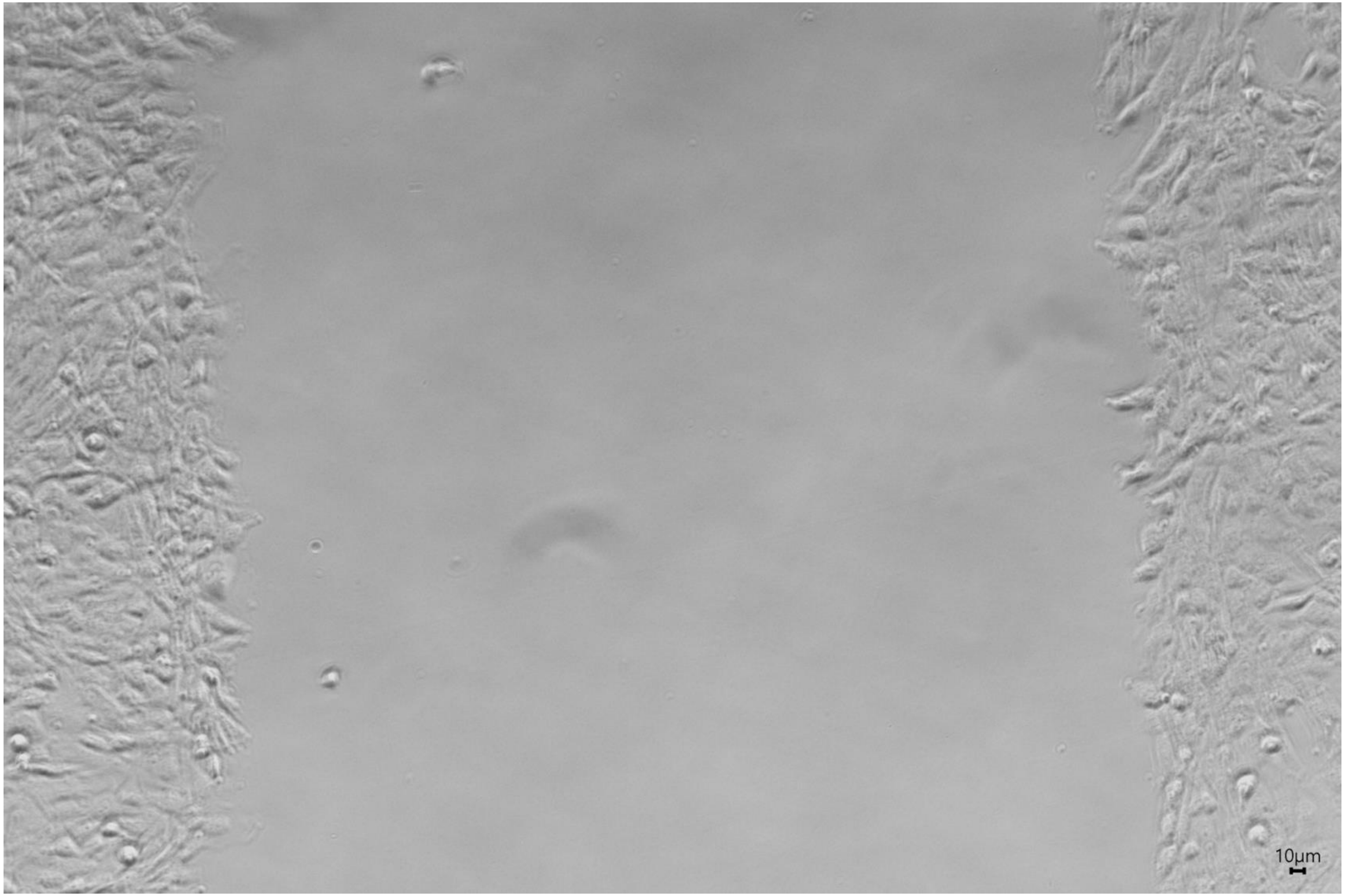

- LRAE 100 μg/mL 18h

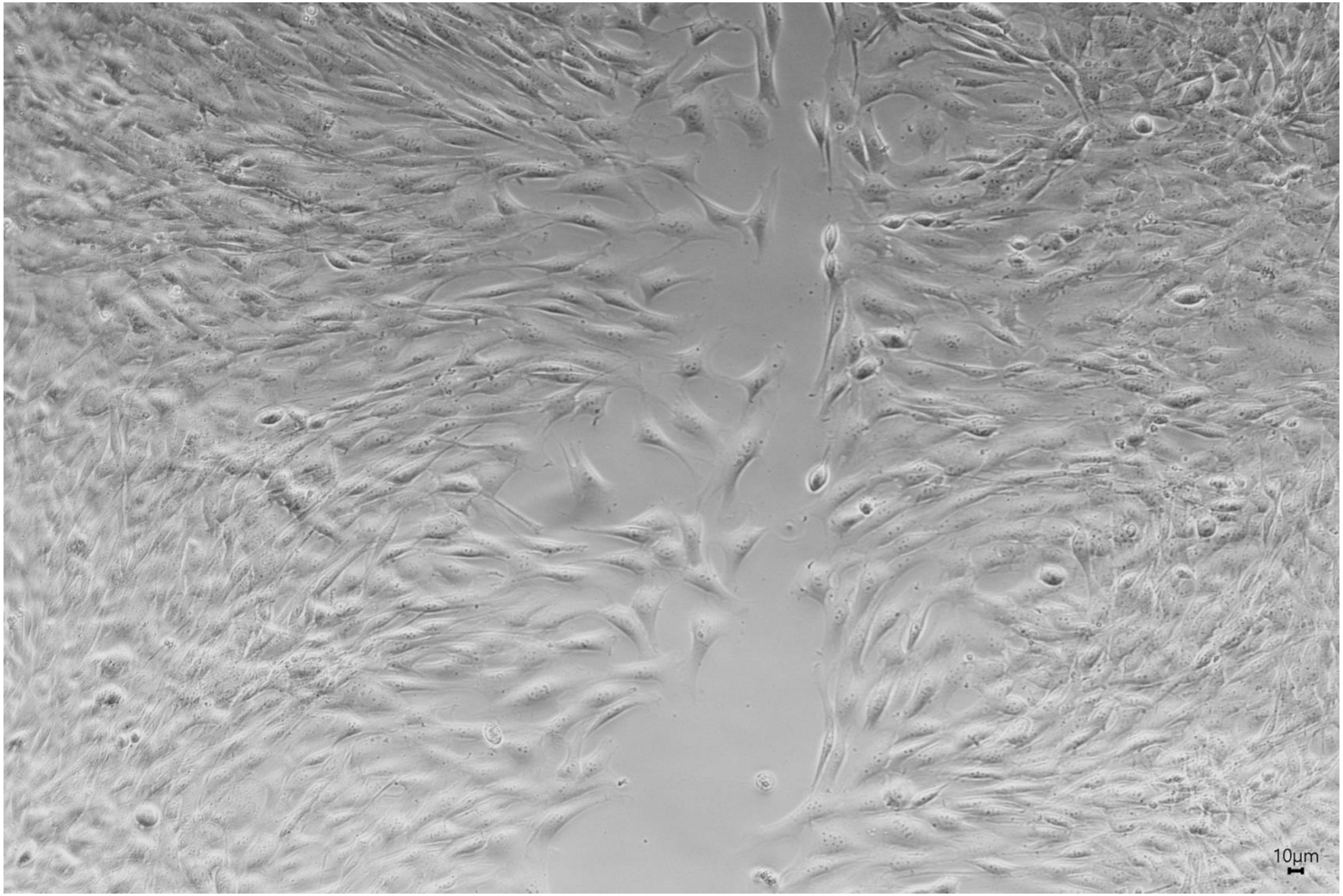

- LRAE 200 μg/mL 0h

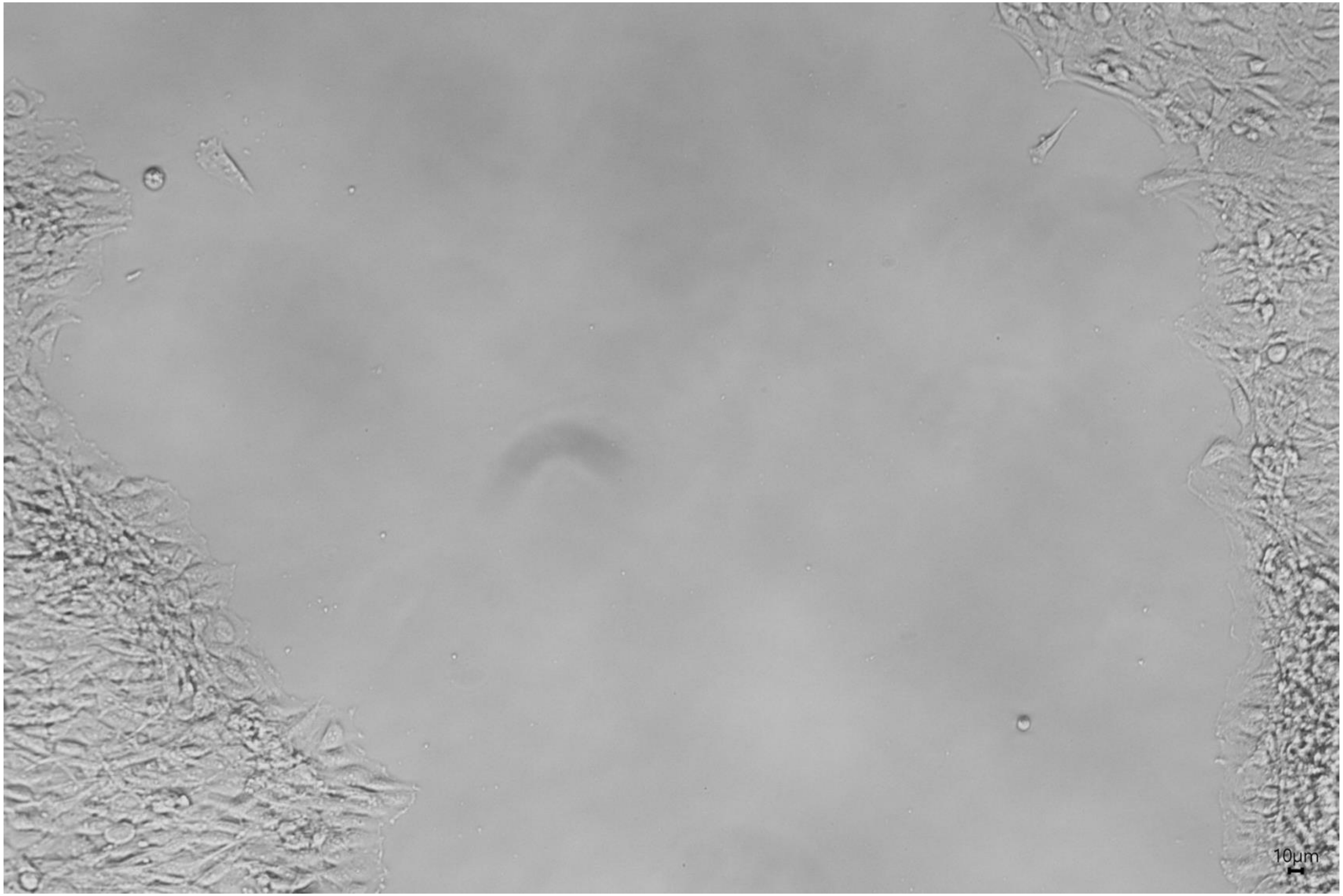

- LRAE 200 μg/mL 18h

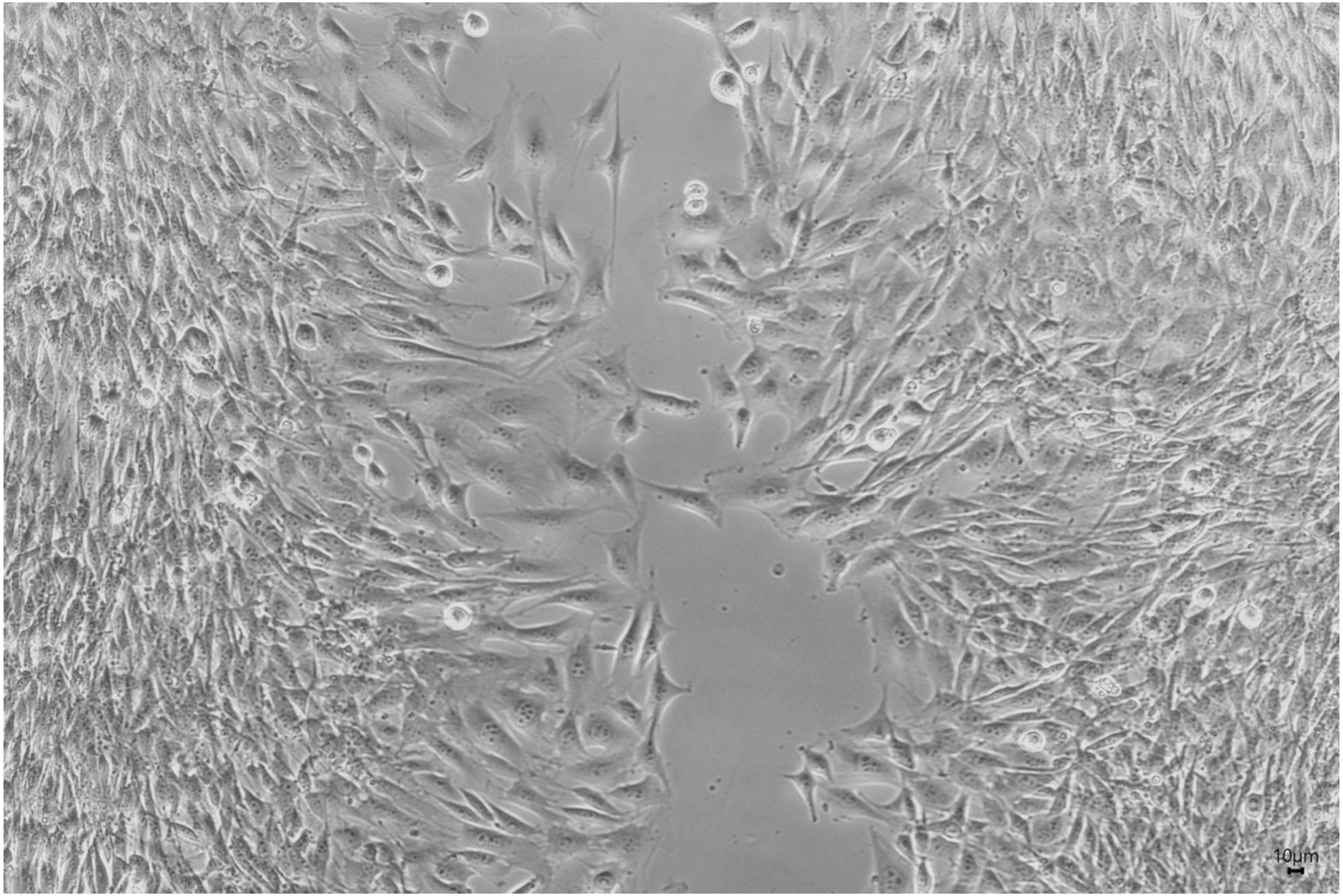

- LRAE 400 μg/mL 0h

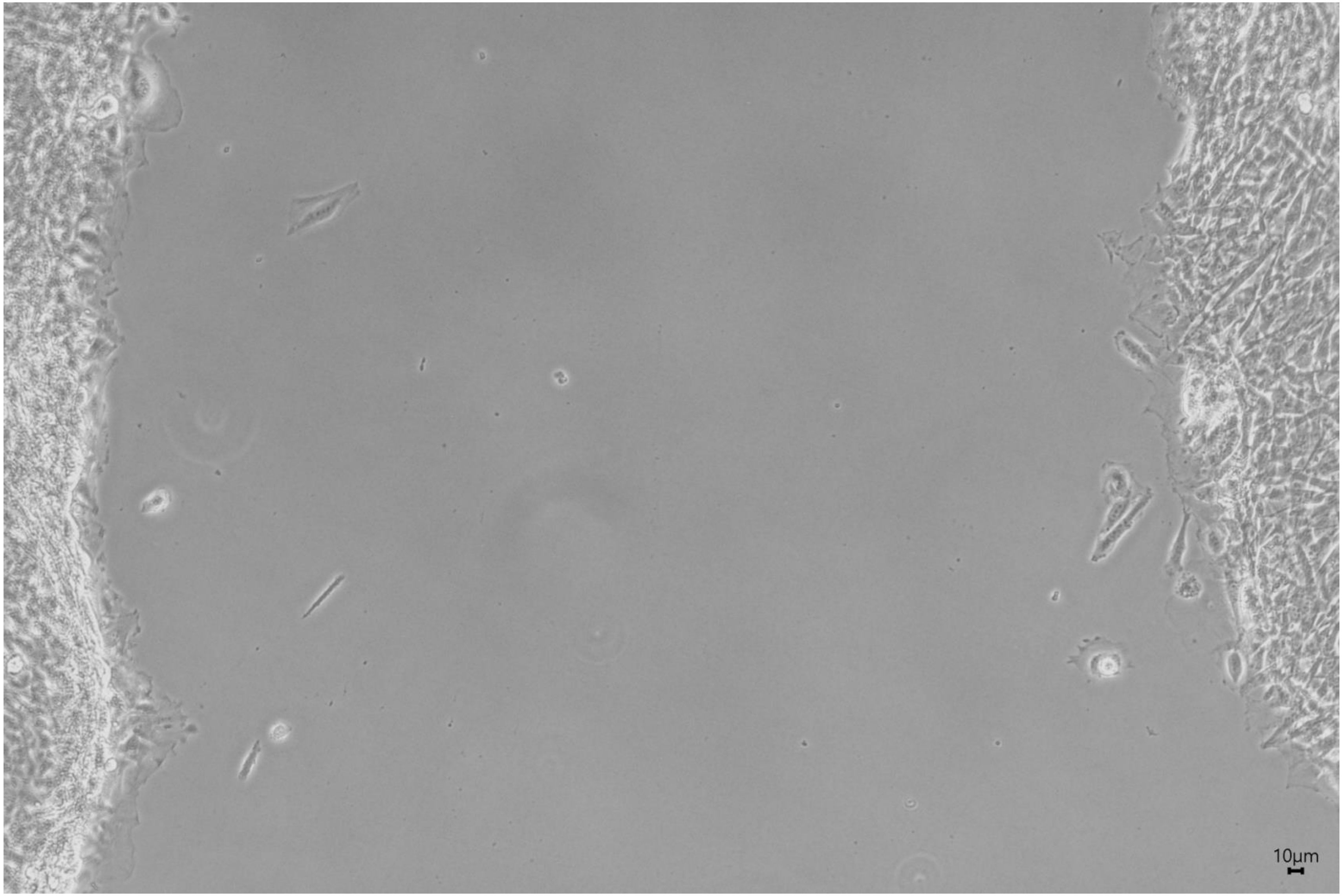

- LRAE 400 µg/mL 18h

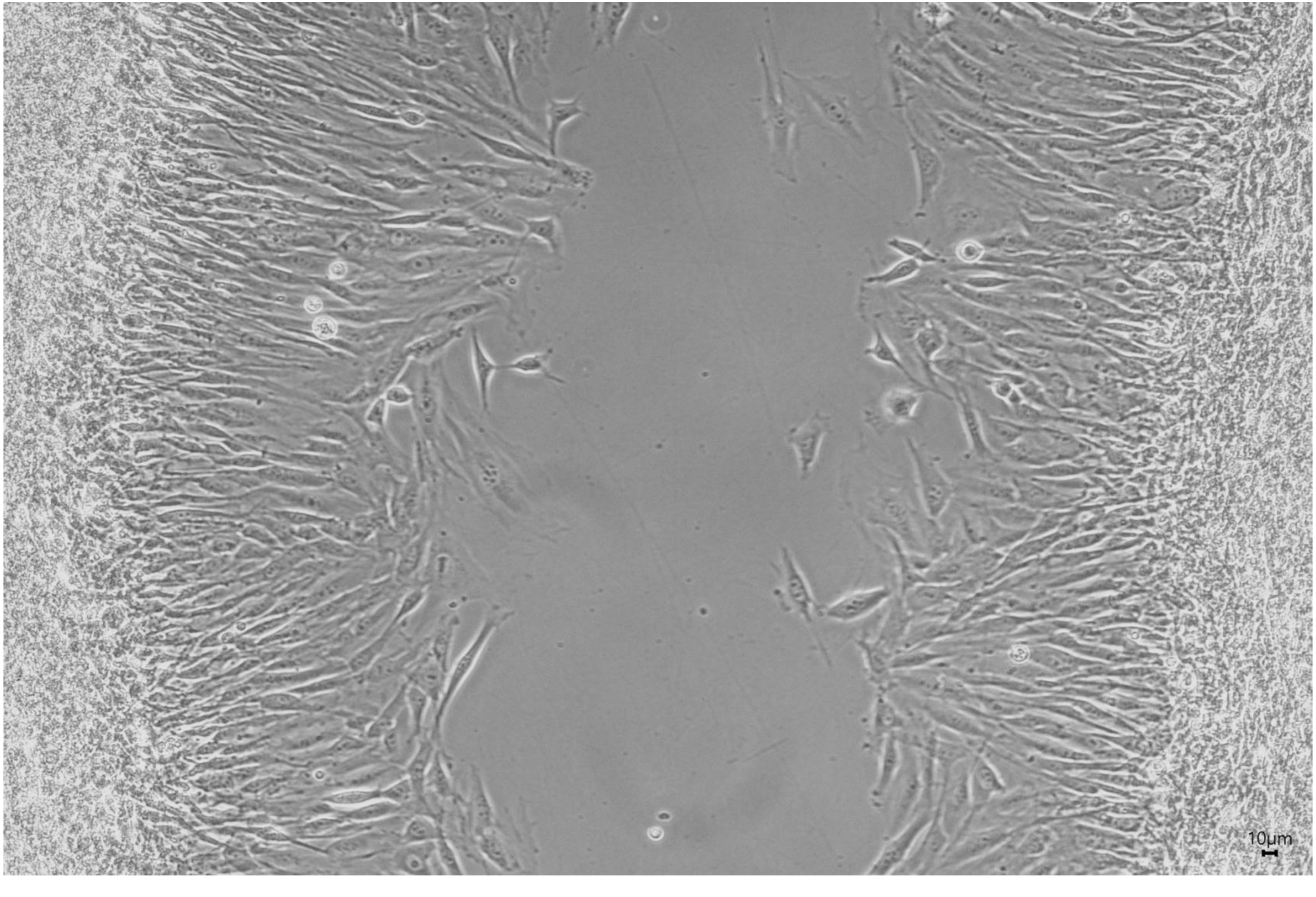

Supplementary material. Table S1. Expanded Table 2.

Authors: González-Vázquez, M., Quílez Guerrero A., Zuzarte, M., Salgueiro L., Alves-Silva, J., De la Puerta R.* *Corresponding author: puerta@us.es

Table 2. Phenolic compounds in aqueous leaf extract of *Lotus rectus* L. (LRAE) confirmed by UHPLC-HRMS/MS

No.	Measured RT (min)	Error (min)	Molecular formula	Expected m/z $[M-H]^-$	Measured m/z $[M-H]^-$	Error (ppm)	Measured MS/MS fragments	Error (ppm)	Attribution	Content mg/g dry extract[1]
1	5.97	-0,0224	$C_{27}H_{30}O_{14}$	577.1563	577.1568	0.8757	117.0344; 183.0452; 255.0301; 285.0405; 430.0905	0.6168; 0.3222; 1.1784; 1.1954; 0.3564	Kaempferol-3,7-O- α-di-rhamnopyranoside	5.60 ± 0.006
2	5.67	-0,0413	$C_{21}H_{20}O_{12}$	463.0882	463.0884	0.3222	227.0356; 243.0301; 255.0301; 271.0252; 300.0278	2.5821; 1.2994; 0.7596; 1.2371; 0.8355	Hyperoside (Quercetin-3-O-galactoside)	0.03 ± 0.001
3	1.1	0,0178	$C_7H_6O_5$	169.0143	169.0142	-0.2156	69.0346; 79.0191; 81.0346; 97.0296; 125.0245	0.2453; 1.5927; 0.2090; -0.2850; 0.8068	Gallic acid	N/A
4	1.98	0,1060	$C_{15}H_{14}O_7$	305.0667	305.0667	-0.0220	109.0296; 125.0245; 137.0245; 167.0351; 219.0663	0.5161; 0.7458; 0.8475; 2.3341; 1.6211	(-)-Gallocatechin	N/A
5	4.04	-0,0099	$C_9H_8O_4$	179.0350	179.0350	-0.0809	89.0398; 107.0503; 134.0375; 135.0454; 179.0353	1.5744; 0.8512; 1.3138; 1.4745; 0.8140	Caffeic acid	< LOQ
6	5.58	0,0248	$C_{27}H_{30}O_{15}$	593.1512	593.1517	0.8077	183.0452; 255.0299; 283.0248; 430.0905; 447.0933	0.9891; 1.1784; 1.2924; 0.9951; 1.1775	Kaempferol-3-O-β-glucopyranoside-7-α-rhamnopyranoside	N/A
7	5.77	-0,0402	$C_7H_6O_3$	137.0244	137.0243	-0.8785	65.0398; 93.0347; 137.0245	1.2169; 0.9201; 1.0703	Salicylic acid	N/A
8	6.06	-0,0259	$C_{21}H_{20}O_{11}$	447.0933	447.0937	1.0171	151.0038; 243.0300; 255.0303; 271.0252; 300.0279	0.3868; 0.6576; 1.4643; 1.4667; 1.24517	Quercitrin (Quercetin-3-O-rhamnoside)	N/A
9	6.82	-0,0179	$C_{15}H_{12}O_5$	271.0612	271.0613	0.1800	65.0033; 83.0140; 107.0139; 119.0503; 151.0038	1.6165; 1.0710; 1.5440; 0.8294; 1.1952	Naringenin	< LOQ
10	8.08	-0,0447	$C_{15}H_{12}O_4$	255.0663	255.0663	0.0313	65.0034; 83.0141; 107.0139; 151.0038; 171.0451	1.6165; 1.6224; -0.0961; -0.2195; 2.8591	Pinocembrin	N/A
11	8.32	-0,0241	$C_{15}H_{10}O_4$	253.0506	253.0506	-0.0455	63.0241; 65.0033; 107.0138; 119.0502; 143.0505	1.4104; 1.3818; 1.3298; 2.6879; -0.6964	Chrysin	N/A

[1]Quantification data are expressed as mean ± standard deviation (n = 3). **Abbreviations**: **No.,** compound number; **RT,** retention time; **LOQ,** limit of quantification; **N/A,** not quantified.
